# Examining the changes in the prevalence of Hepatitis a in Türkiye: systematic review and metaanalysis

**DOI:** 10.1186/s12889-024-20783-4

**Published:** 2024-11-26

**Authors:** Özge Karakaya Suzan, Murat Bektaş, Mustafa Altındiş, Özge Kaya, Ayşe Eroğlu, Serap Çetinkaya Özdemir, Seda Tecik, Ahmet Naci Emecen, Nursan Çınar

**Affiliations:** 1 Department of Nursing, Faculty of Health Sciences, Sakarya University, Esentepe Campus, Serdivan, Sakarya, 54187 Turkey; 2 Pediatric Nursing Department, Dokuz Eylul University, Faculty of Nursing, Izmir, 35340 Turkey; 3 Department of Clinical Virology and Microbiology, Sakarya University Faculty of Medicine, Sakarya, Turkey; 4 Institute of Health Sciences, Sakarya University, Sakarya, Turkey; 5 Department of Public Health, Epidemiology Subsection, Faculty of Medicine, Dokuz Eylül University, Izmir, Turkey

**Keywords:** Hepatitis A virus, Epidemic, Prevalence, Türkiye

## Abstract

**Background:**

The incidence of Hepatitis A is increasing worldwide. Yearly classification of Hepatitis A Seroprevalence for different times in different regions of Türkiye allows indirect measurement of year-specific incidence rates of HAV infection and can be considered the best way to define Hepatitis A status. This study aimed to examine the change in the incidence of hepatitis A by systematically evaluating the available data on the seroprevalence of anti-HAV antibodies in the Turkish population.

**Methods:**

Studies published between First January, 2000- 31 December 2023 that meet the inclusion criteria searched between 16.09.2023 and 31.01.2024 in nine databases (MEDLINE, Web of Science, PubMed, ScienceDirect, Scopus, Eric, CINAHL Complate, TR DİZİN, TÜBİTAK Ulakbim). Independently by two reviewers evaluated all titles and abstracts with consensus-based decision making. The Joanna Briggs Institution (JBI) Analytical Cross Sectional Studies and Prevelance Studies list were used in this study. Pooled prevalence was calculated using 95% confidence intervals. Heterogeneity between studies was assessed by Cochrane’s Q and I2. The random effect model was selected by Cochrane’s Q and I2. Funnel plots were used for publication bias. The data were analyzed via Jamovi 1.2.22.

**Results:**

Data were extracted from 63 studies. Pooled prevalence was calculated using 95% confidence intervals. Funnel plots were used for publication bias. In this meta-analysis, data were obtained from all geographical regions of Türkiye, and the general prevalence of Hepatitis A in the population was found to be 53% [95% CI 0.47, 0.59; I2 = 99.94%]. In subgroup analysis conducted by year, the prevalence of hepatitis A was 45% [95% CI 0.22, 0.67; I2 = 99.8%] between 2002 and 2006, 52% [95% CI 0.39, 0.65; I2 = 99.54%] between 2007 and 2011, 60% [95% CI 0.49, 0.70; I2 = 99.9%] between 2012 and 2016, and 51% [95% CI 0.41, 0.61; I2 = 99.97%] as of 2017. Additionally, a systematic review revealed that vaccination, socioeconomic status, and sex may also affect HAV seroprevalence.

**Conclusions:**

HAV seroprevalence in Türkiye; It was observed that it increased between 2002 and 2016 and decreased until today as of 2017. This systematic review provide a comprehensive overview of HAV virus epidemiology and identify key knowledge gaps, contributing crucial information for influencing factors.

**Trial registration:**

PROSPERO ID = CRD42023464384.

**Supplementary Information:**

The online version contains supplementary material available at 10.1186/s12889-024-20783-4.

## Introduction

Hepatitis A is a viral liver disease caused by Hepatitis A virus (HAV), a virus belonging to the family Picornaviridae of the genus Hepatovirus [1]. Hepatitis A is transmitted via the fecal-oral route, usually through poor personal hygiene, direct contact with infected patients, and consumption of contaminated food or water [1–6].

Clinical symptoms of Hepatitis A vary depending on the age of the infected individual. While the infection is often asymptomatic in children, symptoms such as fever, malaise, jaundice, abdominal pain, hepatitis and hyperbilirubinemia may be observed in adults [2, 4]. While most people infected with Hepatitis A recover completely with lifelong immunity, a very few develop fulminant hepatitis which can be fatal [6]. Diagnosis is made by detection of HAV-specific IgM and IgG antibodies or viral RNA by RT-PCR [4, 7].

Hepatitis A is frequently seen in low-income countries [1]. Due to the increase in international travel in the global age, Hepatitis A may also occur in developed countries. Individuals who use substances, homeless people, prisoners, immigrants, people with a history of risky sexual behavior (homosexuals, sex workers), healthcare workers, people who frequently use blood and blood products, Hepatitis B or Hepatitis C people with chronic liver disease such as cirrhosis are located in the risk group in terms of Hepatitis A [1, 8–10].

To protect against Hepatitis A, hygiene rules must be followed, safe water and food must be used, workers in the food industry must be controlled for infection, if well water or tank water is to be used, the water must be chlorinated and wastewater must be controlled. In addition to all the mentioned preventive measures, vaccination is the mainstay of protection against Hepatitis A and should be done before exposure to infection [10, 11].

As risk groups increase, the incidence of Hepatitis A also increases. The World Health Organization (WHO) stated that they estimated that 7,134 people worldwide died due to Hepatitis A in 2016 [6]. The Center for Disease Control and Prevention (CDC) reported that 44,926 Hepatitis A cases were detected in America between 2016 and 2023, and 424 of them had HAV-related deaths [9]. In the Surveillance Report published by the European Center for Disease Prevention and Control in 2022, it was reported that 4,548 cases of Hepatitis A were detected in 30 EU/EEA (European Economic Area) countries. The countries with the highest notification are listed as Hungary, Croatia, Romania and Bulgaria [12]. When studies on seroprevalence in the world were examined, it was found that 28% of children and adolescents in Northern Thailand had HAV seropositivity [13]. In a study conducted in Iraq, it was stated that the overall seroprevalence of HAV IgG was 68.3%. In this study, seroprevalence in the 5–10 age group was 24.4%; 38.3% in the 11–15 age group; 86.9% in the 16–20 age group; 85.4% in the 21–30 age group; 95.3% in the 31–40 age group; and 89.2% in the > 40 age group, and it has been reported that the main factors associated with Anti HAV IgG positivity are rural residence and male gender [14].

In a study in our country, it was stated that the Hepatitis A seropositivity rate was 64.8%. In this study, it was found that it was 55% between the ages of 0–16, 47% between the ages of 17–30, and 73.5% between the ages of 31–45 [15]. In a study conducted on health interns in our country, it was determined that the Hepatitis A seropositivity rate was 20%, and the year with the highest seropositivity rate was 2019 (26.9%) [8]. When the literature was examined, it was seen that there were seroprevalence studies on Hepatitis A in different regions of Türkiye at different times, but it was seen that there were not enough studies to systematize the data regarding these studies by years. Classifying seroprevalence by year allows indirect measurement of year-specific incidence rates of HAV infection and may be considered the best way to describe the hepatitis A status in a country. Calculating a common seroprevalence may allow identification of conditions that cause Hepatitis A in Türkiye. In addition, it may contribute to Türkiye’s increasing measures in the coming years against Hepatitis A, which emerged as a result of natural disasters (floods, earthquakes) and social events (migration, war) that occurred in the years shown and to the development of health-related strategies and policies to reduce transmission. This study aimed to examine the change in the incidence of Hepatitis A by systematically evaluating the available data on the seroprevalence of anti-HAV antibodies in the Turkish population.

## Research question

Is there a change in the prevalence of Hepatitis A in Türkiye over the years?

### Method

Türkiye; It is a country that connects Southeastern Europe and Asia, has seven regions and 81 provinces, and has a total population of 85 million 372 thousand.

### Design

This systematic review was registered in the International Prospective Register of Systematic Reviews (PROSPERO) (ID = CRD42023464384). PRISMA was used as the reporting guideline [16].

### Search strategy and selection criteria

Nine databases (ScienceDirect, Web of Science, MEDLINE, PubMed, Scopus, Eric, CINAHL Complate, TR DİZİN, TÜBİTAK Ulakbim) were searched.A specific search strategy for the each database was created by using keywords according to Population, Intervention, Comparison, Outcome(s) of interest, and study design (PICOS framework) [17]. Studies published between 01  January, 2000- 31 December 2023 that meet the inclusion criteria searched between 16.09.2023 and 31.01.2024 by researchers (see Supplementary Content File 1). This date range was chosen to ensure homogeneity among the methods diagnosing HAV and to fix the common points in the diagnostic criteria. (see Supplementary Content File 1).

P: 0–18 years old children and adults; I: Hepatit A; C: None; O: Hepatitis A prevalence; S:Descriptive, cross-sectional, prevalence.

The inclusion criteria were as follows: (1) studies published between 2000 and 2023, (2) descriptive study, cross-sectional study, (3) having worked with a reliable method (ELISA, PCR), (4) studies published in Turkish or English, (5) the study sample is from Türkiye. The exclusion criteria were: (1) Studies that included mixed populations and did not clearly report the prevalence for each group, (2) health workers and university health department students (nursing, dentistry, etc.), (3) immigrants, (4) those with chronic diseases (hemodialysis, blood disease, HIV positive, Hepatitis B and Hepatitis C), (5) substance addicts and (6) transplant patients.

### Study selection

**Fig. 1:**
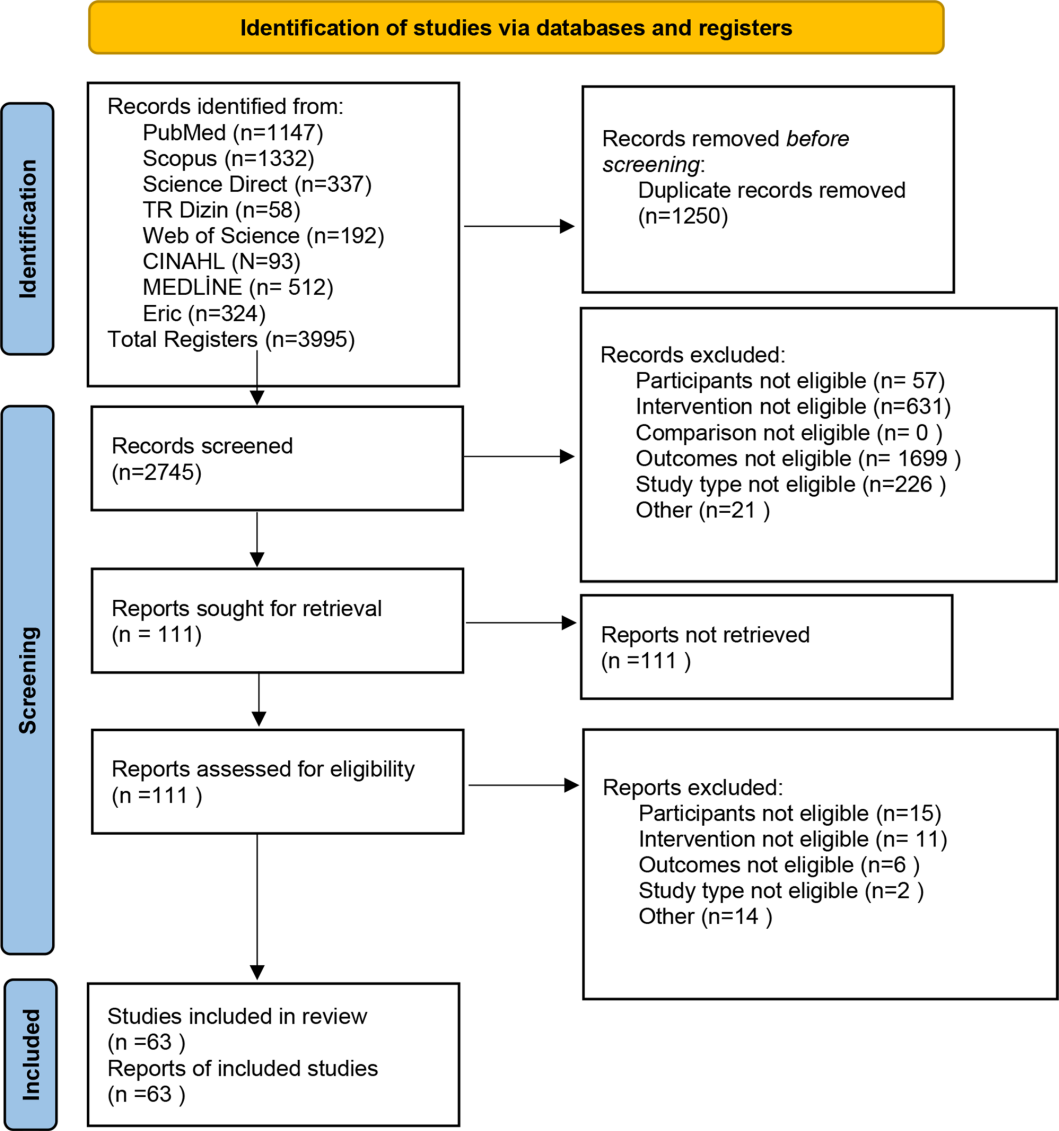
Flow diagram of the selection of studies

Three steps were followed in the selection of studies. These steps include scanning articles, reviewing titles and abstracts, and evaluating full texts. The studies were first scanned by two independent authors who searched the database and duplicates were removed. After duplications were removed, two independent researchers first screened the titles and abstracts and then the full texts for compliance with the inclusion criteria. Any disagreements regarding the inclusion/exclusion of articles were resolved by consensus with a third investigator. For excluded articles, the reasons for exclusion were recorded and reported in the PRISMA flowchart (Fig. 1).

### Data extraction and management

The coding table was used to present the statistical data and characteristics of the study (authors, country, year of publication, sample size, characteristics of participants, etc.) (supplementary file 2). The first and second researchers extracted the data. The third researcher independently checked the accuracy and reliability of the extracted data.

### Assessment of risk of bias compilations

JBI Prevelance Studies (9 items) and Analytical Cross Sectional Studies (8 items) list were used in this study. Critical Appraisal Checklists was developed by the JBI. The scale items are scored as “Yes” = 1 point, “No–Unclear,” and “Not Applicable” = 0 points. A high total score indicates a high-quality study methodology [17, 18].

### Data analysis

JBI quality tools were used to assess the methodological quality of the studies. Inter-rater agreement was analyzed using SPSS 22.0 program and kappa value. The kappa values ​​obtained as a result of the analysis were found to be 0.70 for cross-sectional and prevalence studies, and a high level of agreement was observed between the evaluators [19].

Data were analyzed using Jamovi 1.2.22 and Comprehensive Meta-Analysis (CMA). Pooled prevalence was calculated using 95% confidence intervals. A value of *p* < 0.05 was considered statistically significant. Heterogeneity between studies was assessed by Cochrane’s Q (X^2^ p 0.10) and measured by I^2^. I^2^ statistics with values ​​of 25%, 50%, 75% were considered as low, medium and high heterogeneity, respectively. When I^2^ was below 50% and the cochrane Q value was greater than 0.10, the fixed effect model was chosen, and when it was above 50% and the cochrane Q value was less than 0.10, the random effect model was selected. Funnel plots were used for publication bias.

## Results

As a result of the searches made with the search strategy, a total of 3995 studies were reached. Of these studies, 1250 were removed as a result of duplication and 2634 were removed as a result of title and abstract reviews. The full texts of the remaining 111 studies were examined and 48 studies that did not meet the inclusion criteria were excluded. The selection process of the studies is shown in the flow chart (Fig. 1).

**Fig. 2:**
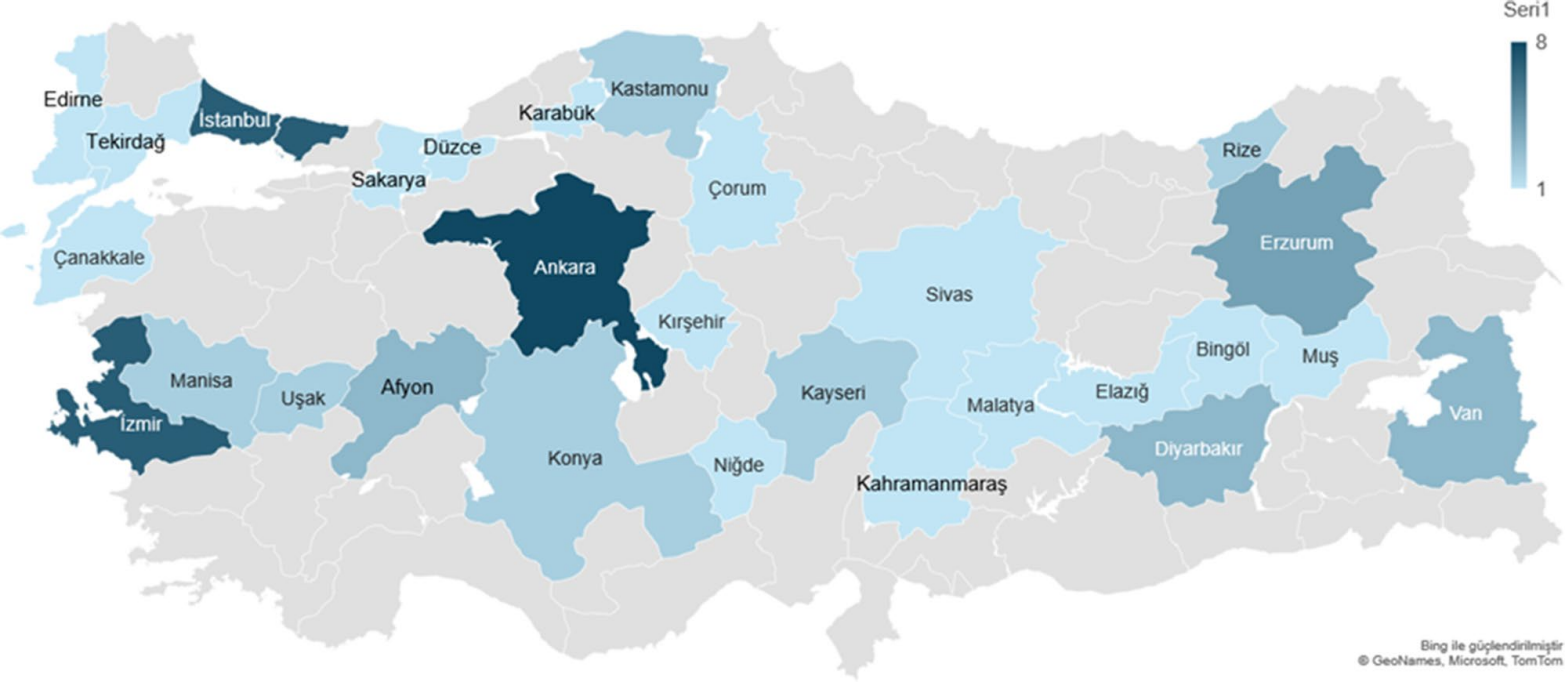
Distribution of included studies by city

**Fig. 3:**
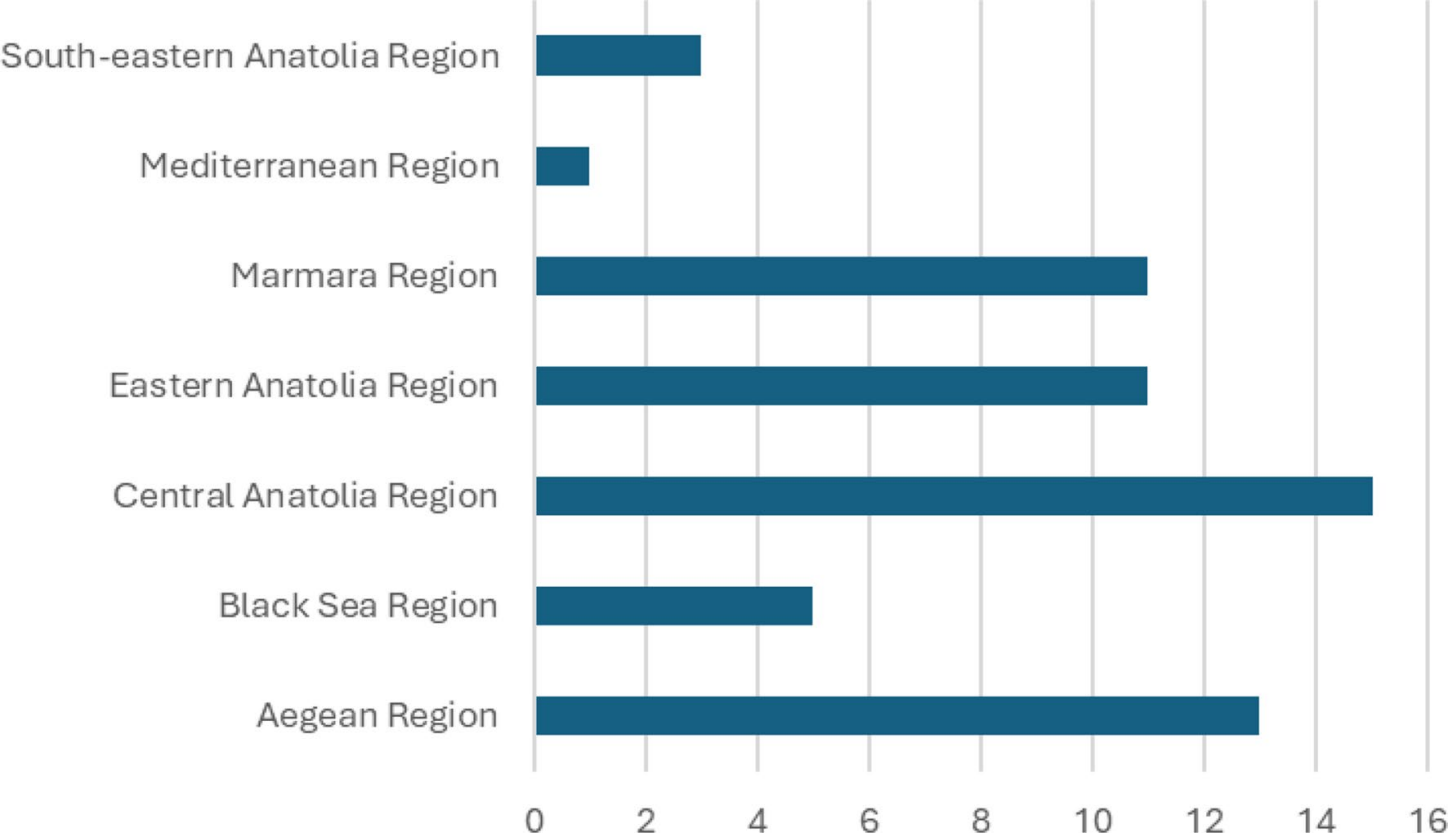
Number of studies included by region

63 studies were included in this meta-analysis study. Hepatitis A seroprevalence in the included studies ranged between 9.4% and 99.1% and sample sizes ranged from 114 to 33,012. 29 studies evaluated were conducted in the pediatric age group, one study was conducted in the adult age group, and 33 studies were conducted in the general population. The distribution of studies included in the meta-analysis by region is shown in Figs. 2 and 3 and is shown in the Supplemental File 2. The data of two studies not included in the figure include more than one region [20, 21].

Homogeneity test results for the studies included in the meta-analysis are as follows. Q value is 278222.696, df value is 62, I^2^ value is 99.94%, τ2 is 0.0654 and p value < 0.001. The results showed that there was significant heterogeneity and average effect sizes were calculated according to the random effects model.

**Fig. 4:**
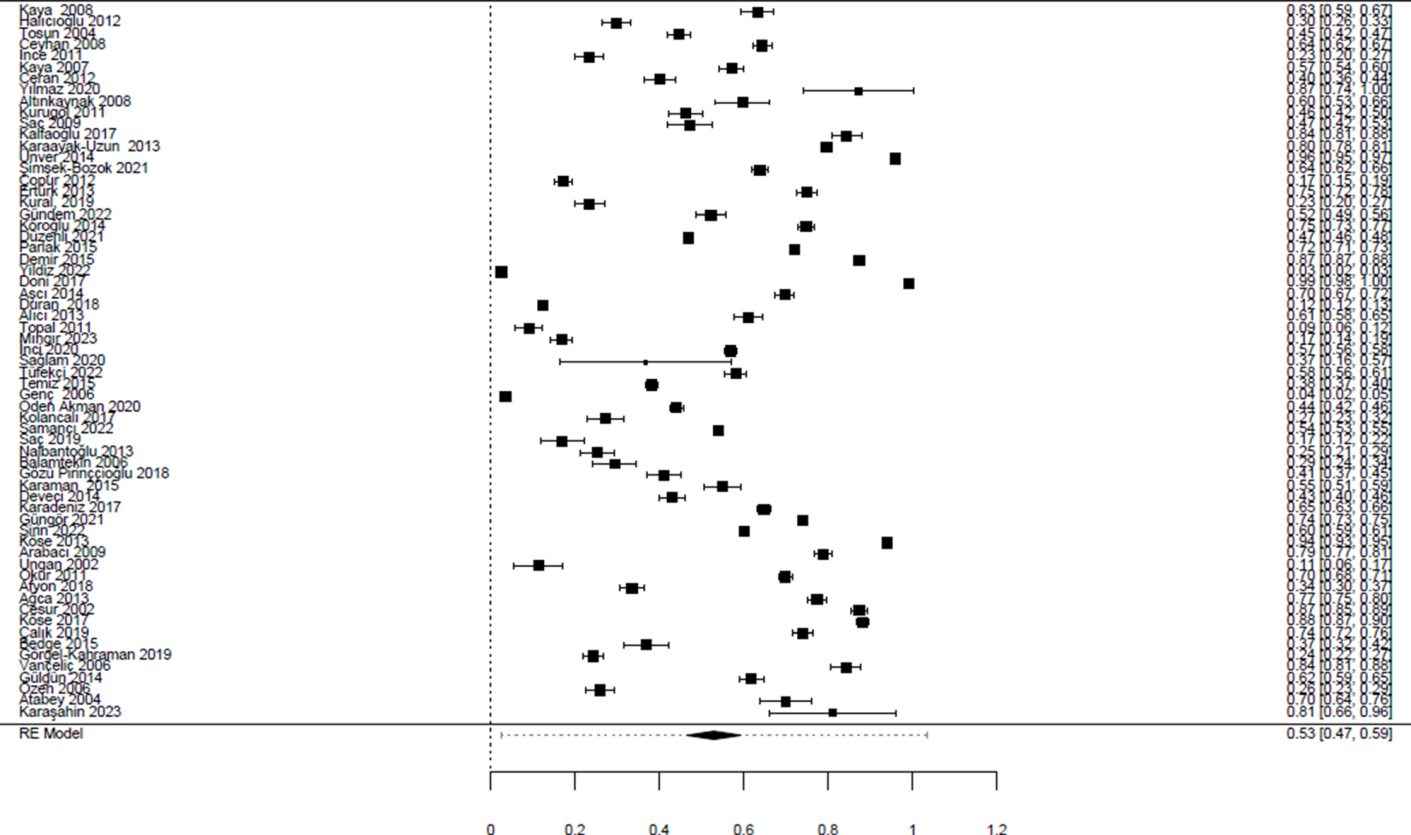
Effect sizes of studies examining hepatitis A prevalence

**Fig. 5:**
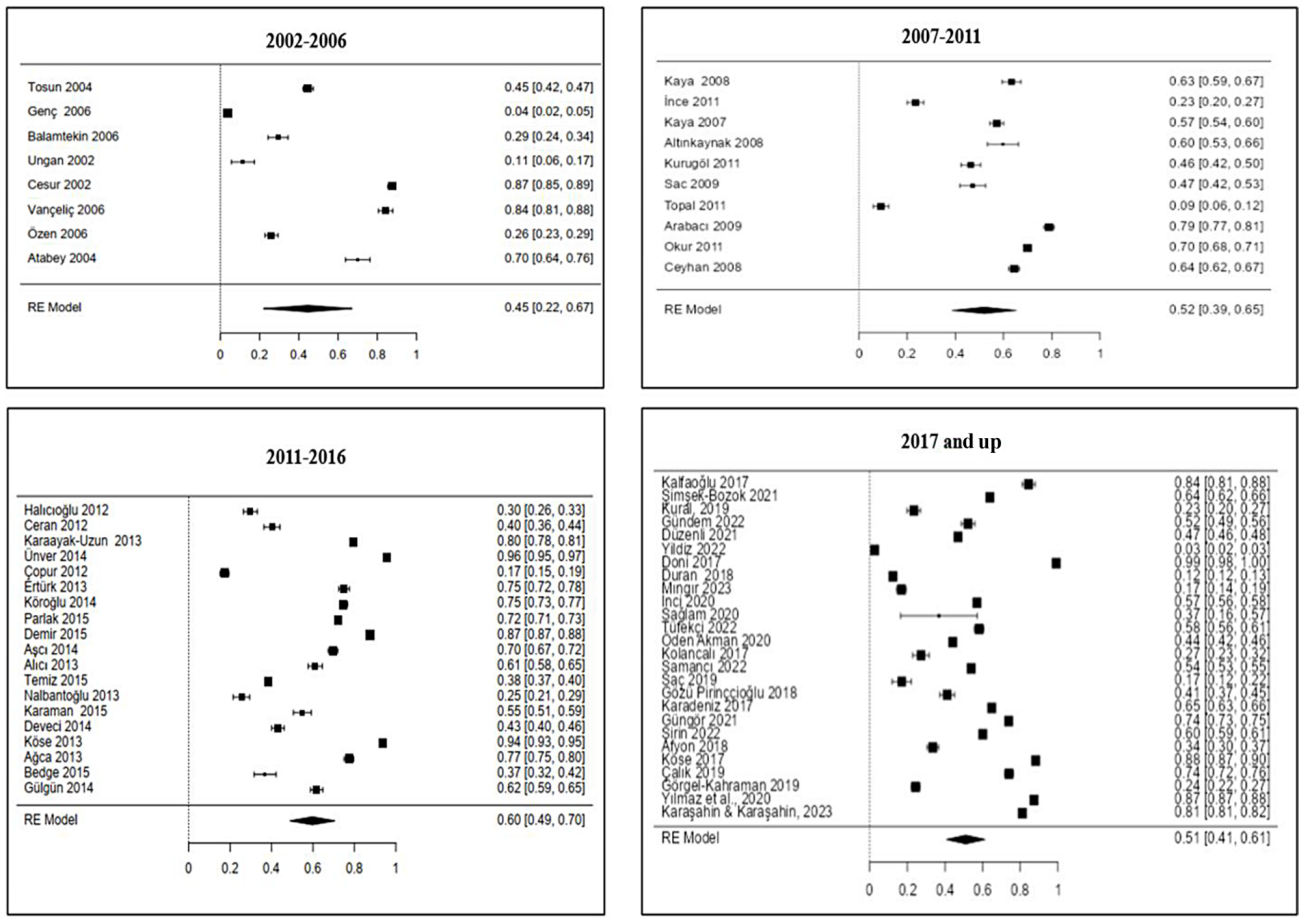
Effect sizes of studies examining hepatitis A prevalence by years

In the meta-analysis, data of 272,092 people from a total of 63 studies were examined [22–84]. In this meta-analysis study, data were found from all geographical regions of Türkiye and the general prevalence of Hepatitis A in the population was found to be 53% [95% CI 0.47, 0.59; *I*^*2*^ = 99.94%] (Fig. 4). In subgroup analyzes conducted by years, the prevalence of Hepatitis A was found to be 45% [95% CI 0.22, 0.67; *I*^*2*^ = 99.8%] between 2002 and 2006, 52% [95% CI 0.39, 0.65; *I*^*2*^ = 99.54%] between 2007 and 2011, 60% [95% CI 0.49, 0.70; *I*^*2*^ = 99.9%] between 2012 and 2016 and 51% [95% CI 0.41, 0.61; *I*^*2*^ = 99.97%] as of 2017 (Fig. 5).

**Fig. 6:**
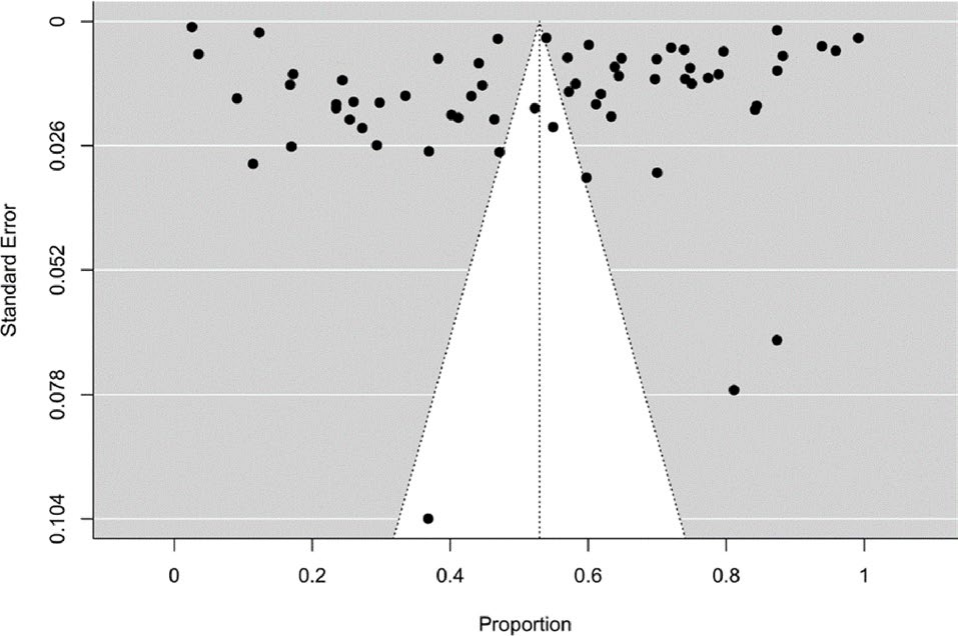
Funnel plot of studies investigating hepatitis A prevalence

Publication bias was evaluated with Begg and Mazumdar Rank correlation, Egger Regression method, Rosenthal’s safe N number and funnel plot methods. According to the Rosenthal error protection coefficient, in order for the effect size to be 0 and to avoid publication bias in the study, there must be more than 325 studies according to the 5 K + 10 formula [85]. K in the formula shows the number of studies included in the meta-analysis. In the analysis, it was determined that 5,321,179 studies were needed for the effect size to be 0, and therefore, there was no publication bias in the study according to the Rosenthal error protection coefficient. While there was no publication bias according to Egger regression analysis (*p* = 0.719), it was determined that there was publication bias according to Begg and Mazumdar rank correlation (*p* < 0.001). Publication bias was examined using the funnel chart given below (Fig. 6).

## Discussion

This systematic review and meta-analysis study presented the general HAV seroprevalence and the change in seroprevalence by years in all geographical regions of Türkiye.

Based on Anti-HAV IgG seropositivity in human serum; geographic distribution areas can be classified as having high, medium and low levels of HAV infection. In the studied populations, seropositivity of < 15%, 15–50%, and > 50% reflects, low, intermediate, and high levels of HAV infection, respectively [86]. It is reported that the HAV seroprevalence range is wide due to socioeconomic differences in various regions of Türkiye [88, 89]. The results of our study are also compatible with the report, and it was observed that the seroprevalence of HAV in Türkiye was between 9.4% and 99.1%. This situation indicates that Türkiye is in the mid-endemic region in terms of HAV infection, in line with the literature [87, 88]. In the seroprevalence study conducted in Cambodia, which is reported to be located in the mid-endemic region, seropositivity was found to be 31.5% and 91.2% in children aged 5–7 years and their mothers, respectively [89].

In this study, data from 63 articles that met the inclusion criteria were examined to determine the seroprevalence of HAV in Türkiye. As a result of the review, the data of 272,092 people from different age groups were meta-analyzed and as a result of the analysis, it was determined that the seroprevalence of HAV in Türkiye was 53%. This rate shows that Türkiye, which was reported as a medium endemic region [90, 91], has shifted to a low endemic region. However, those who are in the risk group for hepatitis A infection; individuals who use substances, the homeless, prisoners, immigrants, those with a history of risky sexual behavior (homosexuals, sex workers), healthcare workers, those who frequently use blood and blood products, those with cirrhosis and chronic liver disease were excluded from the study [1, 5, 8, 10]. Therefore, Türkiye may seem to have shifted to the low endemic zone. This situation should be taken into consideration when evaluating the results of our study.

It is clear that it is important to determine the change in the seroprevalence of the disease over the years in order to take protective measures against HAV infection. In this regard, in order to understand the change in seroprevalence in Türkiye, subgroup analysis was performed according to the publication years of the studies. As a result of the analysis, seroprevalence; It was observed that it was 45% between 2002 and 2006, 52% between 2007 and 2011, 60% between 2012 and 2016 and 51% as of 2017 in Türkiye. Hepatitis A vaccine was added to the routine vaccination calendar in 2012 and is administered in two doses to babies at the end of the 18th and 24th months [92]. The increase in seropositivity after 2012 can be attributed to the fact that it coincided with the beginning of the national vaccination program. Improving living conditions and increasing sanitation are likely to contribute significantly to the change in seroprevalence over the years. Routine childhood vaccination services were disrupted all over the world due to the COVID-19 epidemic in 2020 [93], and a study conducted in Türkiye stated that Hepatitis A vaccine rejection was higher than other vaccines during the epidemic period [94]. The epidemic may have affected the decrease in the Anti-HAV IgG seropositivity rate in Türkiye. In addition, the fact that refugees have to stay in camps where sanitation is inadequate and that they have difficulty accessing clean water and food resources causes the spread of HAV [95, 96]. The inclusion of the refugee population in Türkiye as of 2011 and the increase of this population every year [92] may have caused the change in the prevalence of seropositivity. In addition, it is thought that the natural disaster of heavy rain/flood in some regions of Türkiye in 2017 may have affected the change in seroprevalence [97].

IgG (Anti-HAV IgG) antibody levels produced by the body against the HAV are indicative of developing immunity, and these levels vary depending on vaccination or exposure to Hepatitis A infection [98, 99]. In three different articles we included in the study; the seropositivity rates of vaccinated and unvaccinated children were compared and it was reported that the seropositivity rates of vaccinated children were significantly higher than those of unvaccinated children in both [33, 83, 84]. The effect of vaccination on seropositivity is also emphasized in the literature [100, 101]. However, 19 articles we included in the study provided information about the vaccination status and seropositivity change of the sample [23, 24, 27, 28, 30, 32, 36, 59, 67, 76, 83] the remaining 44 articles did not provide any information on the topic. In future studies, it is recommended to question the vaccination history of the sample, the vaccine dose administered, and evaluate the seroprevalence accordingly.

In some of the studies we included in the systematic review, low socioeconomic status was stated to be a factor that increases the risk of HAV exposure [22, 24, 27, 28, 30, 35, 38, 60, 63, 67]. There are studies in the literature that support this finding and indicate that there is a significant relationship between HAV prevalence and socioeconomic status [102–104].

In the articles we reviewed, there were no significant differences in Anti-HAV IgG positivity between genders [26–28, 30, 31, 33, 40, 46, 52, 70, 79]. In addition, it is seen significantly more frequently in men than in women [29, 42, 50, 51, 54, 68]. There are different studies reporting that it is detected significantly more frequently in women [41, 74]. When the literature was examined, it was seen that there were studies on the incidence rates of Hepatitis A in terms of gender, but these studies gave different results and did not provide sufficient data according to age groups [105–107]. The studies we included also support this situation. Green et al., (2023) [108] in their study, with a better understanding of the differences in HAV incidence rates according to gender; It has been emphasized that the role of genetic, hormonal determinants and gender as a biological variable in HAV infection can be revealed. In studies to be carried out in this direction, it is recommended to provide sufficient data for different age groups in order to better understand the difference in HAV prevalence according to gender.

In the evaluation of publication bias of the systematic review and meta-analysis study; it was determined that there was no publication bias in the study according to the Rosenthal error protection coefficient and Egger regression analysis, but there was publication bias according to the Begg and Mazumdar rank correlation. When we look at the age groups in the studies we analyzed; eight studies published in 2002–2006 and 10 studies published in 2007–2011 consisted mostly of children (75%, 80%, respectively). It is seen that 19 studies published between 2012 and 2016 and 26 studies published as of 2017 mostly consist of the general population (63.2%, 65.4%, respectively) (Supplementary File 2). The sample size in the studies varies between 114 and 33,012. Moreover, although we examined studies from all geographical regions of Türkiye, the number of studies included according to the regions differs from each other and there is heterogeneity among studies. This is a limited aspect of our study and may have caused publication bias in our study. It has been observed that there is a need to conduct studies with high evidence quality, in which large samples are included and seroprevalence data specific to each age group are given separately. In addition, it was observed that there were differences in the classification of age groups between studies, and sufficient data could not be obtained for subgroup analysis specific to age groups. It is recommended to develop standardization in this regard, to make age groupings in line with this standardization and to present detailed data in studies.

When considering the recommendations for protection from Hepatitis A within the framework of global and national prevention programs, it has been observed that; complying with hygiene rules, using safe water and food, ensuring control of infection in workers in the food sector, chlorinating water if well or tank water is used, and ensuring control of wastewater. In addition to the mentioned protective measures, it is reported that vaccination is the main basis for protection from Hepatitis A and should be done before exposure to infection [10, 11, 109]. From this perspective, it is very important to conduct studies to determine the groups at risk for HAV in the world and in Türkiye. In the light of the studies, it is recommended to implement protective measures and vaccinate the groups at risk. In this direction, it is thought that the prevalence of HAV will decrease globally.

## Conclusion

This meta-analysis study presented the general HAV seroprevalence and the change in seroprevalence by years in all geographical regions of Türkiye. Additionally, with the systematic review; it has been observed that vaccination, socioeconomic status and gender may also affect HAV seroprevalence.

HAV seroprevalence in Türkiye; increased between 2002 and 2016 and decreased until today as of 2017. Change in seroprevalence according to years; it is thought that Türkiye’s start to receive immigration in 2011, the Hepatitis A vaccine being added to the vaccination calendar in 2012, and the heavy rain/flood disaster in 2017 may have had an impact. In this regard, it is important for countries to make the necessary policies and plans regarding migration, epidemics and natural disasters.

The heterogeneity among the studies we reviewed can be considered a limitation of our article. In the studies to be carried out in this direction; It is recommended to develop standardization in the classification of age groups, to provide detailed data on HAV prevalence specific to age groups and gender, to question the vaccination history of the sample, the vaccine dose administered, and to evaluate the seroprevalence accordingly.

## Electronic supplementary material

Below is the link to the electronic supplementary material.

Supplementary Material 1

Supplementary Material 2

## Data Availability

Data or information needed to re-produce the findings presented are available from the corresponding author upon reasonable request.
