# Supplementary material for: Examining the changes in the prevalence of Hepatitis a in Türkiye: systematic review and metaanalysis
Source: BMC Public Health. 2024 Nov 26;24:3280. doi: 10.1186/s12889-024-20783-4 (PMC11590238; doi:10.1186/s12889-024-20783-4)
Supplement: Supplementary file 1 — Supplementary Material 1 [file 12889_2024_20783_MOESM1_ESM.docx]

**Detailed search strategy**

| **Key Concepts** | **Keywords** | **MeSH2023 Terms** | **CINAHL Headings** | **Thesaurus** |
| --- | --- | --- | --- | --- |
| **Concept #1**  **Hepatitis A** | - Hepatit* A virus - HAV - Hepatit* A - Hep A - Viral hepatit* A - VHA | - Hepatitis A - Hepatitis, Viral, Human+ | - Hepatitis A | - |
| **Concept #2**  **prevalence** | - Prevalence - Incidence - Seroprevalence | - Incidence - Prevalence - Basic Reproduction Number | - Incidence - Prevalence - Basic Reproduction Number | - [Incidence](javascript:XslPostBack('ctl00$ctl00$MainContentArea$MainContentArea$xslResults','ThesaurusLink','LinkTarget%7CauthorityList%24LinkTerm%7CDE%2B%2522Incidence%2522%24ResultID%7C1');) |
| **Concept #3**  **Turkey\Türkiye** | - Turkey - Türkiye | - Turkey - Middle East+ | - Turkey - Middle East+ | - |
| Keyword-inclusive strings were systematically searched within the title, abstract, and keyword fields across the databases. | | | | |

**Database: SCOPUS**

**Interface – Scopus, Final Search Date: Jan 30^nd^ 2024**

**Search Screen – Advanced Search**

**Terms: Keywords only, controlled vocabulary not applicable**

**Search Strings**

S1 🡪 TITLE-ABS-KEY ( “Hepatit* A virus” OR HAV OR “Hepatit* A” OR “Hep A” OR “Viral hepatit* A”OR VHA) 🡪 *107,834 Results*

*S2*🡪 TITLE-ABS-KEY ( prevalence OR incidence OR seroprevalence ) 🡪7,144,942 Results

*S3*🡪 TITLE-ABS-KEY (Turkey) 🡪1,539,830 Results

S4 🡪 S1 OR S2 AND S3 🡪 *2,323 Results*

**The limitations available at the interface were not used, except for the followings:**

Publication Year: 2000-Current 🡪 2,186 Results

Language: English or Turkish 🡪 2,126 Results

Document Type: Article 🡪 **1,332 Results**

**Database: CINAHL Ultimate**

**Interface – EBSCOhost Research Databases, Final Search Date: Jan 31^nd^ 2024**

**Search Screen – Advanced Search**

**Terms: Keywords and CINAHL Subject Headings Merged**

**Search Strings**

S1 🡪 ( (MH "Hepatitis A") OR ( “Hepatit* A virus” OR HAV OR “Hepatit* A” OR “Hep A” OR “Viral hepatit* A”OR VHA )🡪 *2,948 Results*

*S2*🡪 ( (MH "Incidence") OR (MH "Prevalence") OR (MH "Basic Reproduction Number") OR ( prevalence OR incidence OR seroprevalence )🡪7,144,942 Results

*S3*🡪 (Turkey OR Türkiye) 🡪28,306 Results

S4 🡪 S1 OR S2 AND S3 🡪 *94 Results*

**The limitations offered by the interface are not used, except for the followings:**

Published Date: 2000-2023 🡪 93 Results

Publication Type: Article, Research 🡪 **93 Results**

**Database: MEDLINE**

**Interface – EBSCOhost Research Databases, Final Search Date: Jan 30^nd^ 2024**

**Search Screen - Advanced Search**

**Terms: Keywords and MeSH Terms Merged**

**Search Strings**

S1 🡪(MH "Hepatitis, Viral, Human+") OR (MH "Hepatitis A") OR ( ( “Hepatit* A virus” OR HAV OR “Hepatit* A” OR “Hep A” OR “Viral hepatit* A”OR VHA )🡪 *53,060 Results*

*S2*🡪 ( (MH "Incidence") OR (MH "Prevalence") OR (MH "Basic Reproduction Number") OR ( prevalence OR incidence OR seroprevalence ))🡪674,151 Results

*S3*🡪 (MH "Turkey") OR (MH "Middle East+") 🡪162,583 Results

S4 🡪 S1 OR S2 AND S3 🡪 *545 Results*

**The limitations offered by the interface are not used, except for the followings:**

Published Date: 2000- 2023 🡪 512 Results

Publication Type: Research 🡪 **512 Results**

**Database: ERIC**

**Interface – EBSCOhost Research Databases, Final Search Date: Jan 30^nd^ 2024**

**Terms: Keywords and Thesaurus Merged**

**Search Strings**

S1 🡪( ( “Hepatit* A virus” OR HAV OR “Hepatit* A” OR “Hep A” OR “Viral hepatit* A”OR VHA) 🡪 *274 Results*

*S2*🡪 DE "Incidence" OR (prevalence OR Incidence OR Seroprevalence) 11,967 Results

*S3*🡪 (Turkey OR Türkiye) 🡪26,857 Results

S4 🡪 S1 OR S2 AND S3 🡪 *403 Results*

**The limitations offered by the interface are not used, except for the followings:**

Published Date: 2000- 2023 🡪 324 Results

Publication Type: Research 🡪 **324 Results**

**Database: ScienceDirect**

**Interface – ScienceDirect, Final Search Date: Jan 30^nd^ 2024**

**Search Screen – Advanced Search**

**Terms: Keywords only, controlled vocabulary not applicable**

*Wildcards '*' are not supported, in accordance, terms were completed. Only 8 Boolean operators are allowed to use, in accordance, strings were divided, records were then saved separately.*

**Search String**

Find articles with these terms *( (“Hepatitis A virus” OR “Hepatitis A” OR “Hep A”) AND (prevalence OR Incidence) AND (Turkey))* 🡪 1,543 Results

**The limitations offered by the interface are not used, except for the followings:**

Custom Year Range: 2000 – 2023 🡪 1,287 Results

Article Type: Research Articles, case report 🡪 337 Results

**Database: Web of Science Core Collection, All Editions**

**Interface – Web of Science, Final Search Date: Jan 30^nd^ 2024**

**Search Screen – Advanced Search**

**Terms: Keywords only, controlled vocabulary not applicable**

**Search Strings**

S1 🡪 TS=( ( “Hepatit* A virus” OR HAV OR “Hepatit* A” OR “Hep A” OR “Viral hepatit* A” OR VHA) 🡪 *18,500 Results*

*S2*🡪 TS=(( prevalence OR incidence OR seroprevalence ) )🡪2,077,909 Results

*S3*🡪 TS=((Turkey OR Türkiye) )🡪164,718 Results

S4 🡪 S1 OR S2 AND S3🡪 *192 Results*

**The limitations offered by the interface are not used, except for the followings:**

Custom Year Range: 2000 – 2023 🡪 215 Results

Web of Science Index: Excluded: review article, meeting abstract, book chapter, letter 🡪 **185 Results**

**Database: Pubmed**

**Interface – Pubmed, Final Search Date: Jan 30^nd^ 2024**

**Search Screen – Advanced Search**

**Terms: Keywords and MeSH Terms Merged**

**Search Strings**

S1 🡪 ("Hepatitis A"[Mesh]) OR "Hepatitis, Viral, Human"[Mesh] OR ( ( “Hepatit* A virus” OR HAV OR “Hepatit* A” OR “Hep A” OR “Viral hepatit* A” OR VHA )🡪 *158,618 Results*

*S2*🡪 (("Prevalence"[Mesh] OR "Basic Reproduction Number"[Mesh] OR "Incidence"[Mesh]) OR (Seroprevalence))🡪649,473 Results

*S3*🡪 (("Middle East"[Mesh]) OR "Turkey"[Mesh]) OR Türkiye))🡪342,323 Results

S4 🡪 S1 OR S2 AND S3 🡪 *1327 Results*

**The limitations offered by the interface are not used, except for the followings:**

Date: 2000 – 2023 🡪 1149 Results

**From Other Sources**

**Search Engine: Tr Dizin**

**Interface – Tr Dizin, Final Search Date: Jan 30^nd^ 2024**

**Search Screen – Advanced Search**

**Terms: Keywords, controlled vocabulary not applicable**

**Search Strings**

**All field** 🡪 (("hepatitis A") AND ("prevalence" OR "incidence" OR "seroprevalence") AND ("Turkey" OR "Türkiye"))🡪58 Results

**Access type selected as everything.**
