# Supplementary material for: Examining the changes in the prevalence of Hepatitis a in Türkiye: systematic review and metaanalysis
Source: BMC Public Health. 2024 Nov 26;24:3280. doi: 10.1186/s12889-024-20783-4 (PMC11590238; doi:10.1186/s12889-024-20783-4)
Supplement: Supplementary file 2 — Supplementary Material 2 [file 12889_2024_20783_MOESM2_ESM.docx]

**Supplemental Digital Content 2, Table 1:** Coding Table

| **Authors, Year, Region** | **Design, N,n** | **Study Aim** | **Population** | **Year of**  **Screening** | **Laboratory Testing Technique** | **Anti-HAV IgG**  **prevalence (%)** | **95% CI** | **Results showing the relationship between demographic characteristics and prevalence** | **Vaccination %** | **Quality** |
| --- | --- | --- | --- | --- | --- | --- | --- | --- | --- | --- |
| **Kaya et al. (2008)[22], Marmara Region, Düzce** | Prospective study  N:589  Group 1 (ages 6 months to 5.9 years); n = 127,  Group 2 (ages 6.0– 12.9 years); n = 100)  Group 3 (ages 13.0–17.0 years); n = 362 | Sero-epidemiological data about both  infections showed higher prevalence rates soon after the 1999 earthquakes in Duzce, Turkey.  The aim of the present study was to evaluate the data 4 years after the earthquakes. | **Age:** 6 months to 17 years (mean 11.5$\pm$ 5.4 years)  **Gender (%):**  Girls: 27.7%,  Boys: 72.3% | April to June 2003 | Enzyme-linked immunosorbent assay | **Anti-HAV IgG (n,%):** 373  63.8% |  | **Age**  Group 1: 28.8%  Group 2: 79%  Group3: 76.8%  **Economic condition:**  **Poor:**  **Anti-HAV (+):418** (82.9%)  P < 0.01  **Adequate:**  **Anti-HAV (+):**22 (25.8%)  P < 0.01 | - | **6/9** |
| **Halicioglu et al., (2012)[23], Aegean region, İzmir** | Cross-sectional study  N: 729 | This study was aimed to determine the age-specific pattern of the prevalence and demography of HAV infection in a pediatric and adolescent outpatient clinics in Izmir. | **Age**  8.5$\pm$ 4.2  years (1-18 years.  **Gender**  Female:323  Male:406 | April, 2009 and  December, 2009 | Chemiluminescent Immunoassay on a LIASON instrument. | **HAV IgG** (n, %): 217 29.8% |  | **Age groups**  **<10:** 104 (21.6) **p<0.001**  **>10:** 110 (44.3)  **OR (95%, CI):** 2.9 (2.1e4.0)  **p<0.001** | ① All unvaccinated children against HAV were grouped. | **6/8** |
| **Tosun et al., (2004)[24], Aegean region, Manisa** | -  N:1395 | The aim of the present study was to evaluate the seroprevalence of the HAV infection in children and adolescents in Manisa, Turkey, and to verify whether the increased incidence of HAV infection in other parts of the world and Turkey generally is also true for the area of Manisa. | **6–23 months** n:272  **2–6 years** n:389  **7–10 years** n:288  **11–14 years** n:271  **15–17 years** n:175 | January 2000 and June 2001 | Enzyme-linked immunosorbant assay: micro EIA method | **Anti HAV IgG (n, %):** 623  44.6% |  | **6–23 months (n:272):** 130 (47.8%)  **2–6 years (n:389):** 92 (23.7%)  **7–10 years (n:288):** 125 (43.4%)  **11–14 years (n:271):** 142 (52.4%)  **15–17 years (n:175):** 134 (76.6%)  ① Total anti HAV seropositivity began to increase after 7 years of age in our study and this was statistically significant (χ2 = 149, P ≤ 0.0001).  **Middle or high socio-economical level:** 213 (34%)  χ2 = 97.3, P = 0.000  **Low socio-economic level:** 410 (66%) | ① None of the cases had been vaccinated against hepatitis A virüs. | **5/9** |
| **Ceyhan et al., (2008)[25], All regions in Turkey** | -  N:1773 | To determine hepatitis A seroprevalence in different regions of Turkey | **Age (years):** 19.3 ± 17.1 (range 1 month–91 years)  **Gender:**  A female to male ratio of 0.9:1. | 1 June 2005 and 31 May  2006 | ELİSA | **anti-HAV IgG**  1142 (64.4%) |  | **South-eastern and Eastern regions**  **5–9 age:** over %80  **After 14 age:** over 90%  **Agean**  **5–9 age:** 36%  **Until 25-29 age:** under 80%  **Marmara regions**  **5–9 age:** 44%  **Until 25-29 age:** under 80%  **Central Anatolia**  **5–9 age:** 34%  **40 to 60 age:** over 72%  **up to 60 age:** less than 80% |  | **7/9** |
| **İnce et al., (2011)[26], Central Anatolia Region, Ankara** | -  N:601 | Hepatitis A seroprevalence among infants aged 12 months in Ankara | **Gender**  **Female:**282  **Male:**319 | August 2007 and  February 2009 | ELISA | **Anti-HAV IgG (n,%):** 141  23.5% |  | **Male: 22.6%**  **Female: 24.5%** |  |  |
| **Kaya et al., (2007)[27], Mediterranian Region, Kahramanmaraş** | -  N:1142 | We investigated HAV seroprevalence and its association with sociodemographic factors among children of various ages in the Eastern Mediterranean region of Turkey. | **Gender**  **Male:** 603  **Female:** 539 | September–December 2005 | ELISA | **anti-HAV IgG (n, %):** 653  57.2% |  | **Age**  **6–23 months:** 35.5%  **2–5 years:** 19.2%  **6–10 years:** 74.3%  **11–14 years:** 83.0%  **15–18 years:** 92.8%  **(P < 0.001)**  **Gender**  **Odds Ratio (CI 95%):**  **Female: 1.00**  **Male:** 1.11 (0.83-1.48)  **p= 0.477**  **Age Odds Ratio (CI 95%):**  **6-23 months:**1.00  **2-5 years:** 0.43 (0.29-0.65)  **p= 0.000**  **6-10 years:** 5.14 (3.39-7.78)  **p= 0.000**  **11-18 years:** 7.18 (4.63-11.08)  **p= 0.000**  **Socioeconomic status Odds Ratio (CI 95%):**  **High:** 1.00  **Middle:** 1.51 (1.02-2.23)  p= 0. 038  **Low:** 2.18 (1.36-3.50)  p= 0.000  **Very low:** 2.10 (1.26-3.44)  p= 0.004 | ① A vaccination history were excluded from the study |  |
| **Ceran et al., (2012)[28], Marmara Region, İstanbul** | Prospective study  N:630 | The aim of this study was to determine the prevalence of hepatitis A and evaluate the associated demographic features in children and young adults in Istanbul | **Gender**  **Female:** 402 (63.8%)  **Male:** 228 (36.2%) | 2007 | ELISA | **Anti-HAV IgG (n,%):** 253  40% | **Age**  (5–9 years): p: 0.0001  **Odds ratio:**  14.095  **95% CI**  7.375 26.935  **Age**  (10–14 years)  p: 0.0001  **Odds ratio**:  4.62  **95% CI**  2.748 7.768  Age  (15–19 years)  p: 0.002  **Odds ratio**:  2.245  **95% CI**  1.361 3.702 | **Age group and seropositivity**  **5–9 years:** 17 (11.4%)  **10–14 years:** 47 (29%)  **15–19 years:** 80 (49.7%)  **20–24 years:** 109 (69%)  **p= 0.0001**  **Gender**  **Female:** 166 (41.3%)  **Male:** 87 (38.2%)  **p:0.440**  **Socioeconomic status**  **Adequate:** 107 (34.4%)  **Inadequate:** 146 (45.8%)  **p: 0.014** | ① Subjects without a history of active immunization against  hepatitis A | **6/9** |
| **Yılmaz et al., (2020)[29], Eastern anatolia region Erzurum,** | Retrospective  N:25.007 | The aim of the study is to investigate the seropositivity of antihepatitis A virus (HAV) IgG and IgM in all age groups in Erzurum | **Age (years)**  28.6±13  (0-93)  **Gender**  **Female:** 14,089 (56.3%)  **Male:** 10,918 (43.7%) | January 2015 – December 2018 | ELISA | **Anti-HAV**  **IgG**  87.3% (21,842) |  | **HAV IgG(+)**  **n (%)**  **0–4:** 107**(**87%)  **5–9:** 793 **(**73.2%)  **10–14:** 1.078**(**58.7%)  **15–19:** 2.138**(**75.2%)  **20–24:** 4.074**(**86.1%)  **25–29**: 4.416(89.8%)  **30–39: 4.842(**96.1%)  **40–49: 2.285(**99.1%)  **50–59: 1.228(**99.1%)  **>60 years: 881(**99.3%)  **p< 0.05**  **Gender**  **Female:** 12,179 (86.4%)  **Male:** 9,663 (88.5%)  p <0.001 |  | **8/9** |
| **Altınkaynak et al., (2008)[30], Eastern anatolia region Erzurum,** | N:226 | The aim of this study was to determine the prevalence rate of hepatitis A virus (HAV) and the socio-epidemiological factors affecting HAV among children aged 0-18 years in Eastern Turkey. | **-** | January and May 2002. | ELISA | **Anti-HAV IgG (n,%):**135  59.7% | **Male:** 48.8-71.8  **Female:** 47.0-71.0  **<1:** 27.1-76.1  **1-3:** 28.0-68.0  **4-6:** 31.1-71.1  **7-9:** 47.7-87.7  **10-12**: 50.0-89.6  **13-15:** 65.3-97.7  **16-18**:36.1-100.0  **Socio economic status**  **High:** 9.6-70.4  **Middle:** 5.9-58.9  **Bad:** 43.6-78.0  **Worst:** 63.0-82.3 | **Gender**  **Male:** 51.3%  **Female:** 48.7%  **Odds Ratio (CI 95%):** 1.05 (0.62-1.79)  **p:0.848**  **Age**  **<1:** 16 (51.6%)  **1-3:** 24 (48%)  **4-6:** 24 (51.1%)  **7-9:** 21 (67.7%)  **10-12**: 21 (70%)  **13-15:** 22 (81.5%)  **16-18**: 7 (70%)  **Odds Ratio (CI 95%):** 1.30 (1.11-1.53)  **p: 0.042**  **Socio economic status**  **High:** 10 (40%)  **Middle:** 12 (32.4%)  **Bad:** 31 (60.8%)  **Worst:** 82 (72.6%)  **Odds Ratio (CI 95%):** 1.01 (0.63-1.61)  **p:0.000** | ① Only two of the 226 children had received the HAV vaccine.  ② The percentage of HAV IgG seropositivity of unvaccinated children was 59.3% | **7/9** |
| **Kurugöl et al., (2011)[31], Aegean region İzmir,** | -  N: 595 | The aim of the present study was, therefore, to determine the current agerelated anti-HAV seroprevalence and to compare it with previously reported prevalence data from the same population and to evaluate the epidemiological shift in HAV serostatus in Izmir, Turkey | **Gender**  **Males:** 236  **Females:** 359  **Age**  0-19 years | February and July 2008 | ELISA | **anti-HAV IgG**  46.4% | **Adjust OR (95% CI)**  **1–4:** Ref  **5–6:** 2.4 (0.5–10.6)  **7–9:** 9.5 (2.4–36.5)  **10–14:** 8.3 (2.2–31.7)  **15–19**: 13.4 (3.6–49.6)  **20–29:** 189.2 (45.7–782.2)  **30–39:** 828.3 (148.5–4618)  **>40:** 3241 (309–33,960) | **Age n (%)**  **1–4:** 71 (4.6%)  **5–6:** 75 (10.3%)  **7–9:** 75 (22.1%)  **10–14:** 76 (23.2%)  **15–19**: 73 (36.4%)  **20–29:** 74 (85.3%)  **30–39:** 75 (95.6%)  **>40:** 76 (99%)  **p < 0.001**  **Gender**  **Male:** 236 (34.7%)  **Adjust OR (95% CI): Ref**  **Female:** 359 (54%)  **Adjust OR (95% CI):** 1.3 (0.7–2.2)  **Family income (monthly)**  **Medium or high:** 338 (31.5%)  **Adjust OR (95% CI): Ref**  **Low:** 257 (57.7%)  **Adjust OR (95% CI):** 4.8 (2.6–8.8) | ① Only people who had not received the hepatitis A vaccine were included in the study. | **7/9** |
| **Sac et al., (2009)[32], Central Anatolia Region Ankara,** | Cross-sectional study  N:335 | The objective of the present study was to determine the seroprevalence of hepatitis A and patient demo-graphics in children between 1 and 15 years old who were admitted to a pediatric outpatient clinic in Ankara, Turkey. | **Age mean, years):** 7.9 ± 2.1  **Gender**  **Female:** 47.5% (159)  **Male:** 52.5% (176) | August – September 2002 | ELISA | **anti-HAV IgG (n,%):** 158  47.2%66 |  | **anti-HAV IgG**  **1– 2:** 15 (34.1%)  **3 – 5:** 15 (23.1%)  **6 – 10:** 69 (48.9%)  **11 – 15*:** 59 (69.4%) |  | **6/8** |
| **Samancı & Akdeniz,**  **(2022)[33], South eastern anatolia region,**  **Diyarbakır,** | Retrospective  N:34.809  before September 2012 **n1**: 14 983 (%43)  after September 2012 **n2:** 19 826 (%57) | The aim of the study was to examine and compare hepatitis A seropositivity in children. | **Age**:0-18  **Age mean (years):** 8,2 ± 5,3  **Gender**  **Female:** 14.698 (%42,2)    **Male:** 20.111 (%57,8) | January 2009-December 2018 | macro enzyme-linked immunosorbent assay method (Architect, Abbott Diagnostics, Abbott Park, Ill, USA) | **anti-HAV IgG (21.267 patient)**  11.467 %53,8  1.group %42,1  2.group %81,9  p: <.001 | - | **Anti-HAV IgG+ Age mean** 7.3 ± 4.4 years  **female:** % 45.5  **male:** % 54.5  p = .029  ① Anti HAV-IgG positivity was significantly higher in group II compared to group I (81.9% vs. 41.2%, P < .001) |  | **6/9** |
| **Saç et al.,**  **(2019)[34], Central Anatolia Region**  **Ankara,** | Retrospective, cross-sectional study  N:320  n: 206 | This study aimed determining the seroprevalence of these infections in Turkish adolescent nursing students. | median age of 17 years  nursing studesnts | September 2014 and September 2015 | Hepatitis A antibody was investigated by chemiluminescence micro particle enzyme immunologically test method (Abbott, Architect, Germany). | **anti-HAV IgG n(%)**  35(%17,0) | - | ① No relation was detected between age and anti-HAV IgG positivity (p=0.187) | ① 14 students vaccinated  Anti-HAV IgG was positive in 30 (15.6%) of the students who did not receive Hepatitis A vaccination. (p=0,067). | **6/8** |
| **Nalbantoğlu et al., (2013)[35], Marmara Region,**  **Tekirdağ,** | Prospective, cross-sectional study  N:452 | This study was designed to determine the current age-related hepatitis A virus (HAV) seroprevalance, vaccination status of children and to evaluate the epidemiological shift in HAV serostatus living in Tekirdağ, which is located in Thrace region, the European part of Turkey. | **Age:** 6 months-12 year  **Age mean:** 6,9±2,2 years  **Male:** 217 (%48)  **Female:** 235 (%52) | March-November 2010 | HAV IgM and IgG collectively with enzyme-linked immunosorbent assay using Vitros ECI Q J&J Company Ortho Clinical Diagnostic Operator. | **HAV IgG n(%)**  115 (25,4) | - | **HAV IgG n(%)**  6 m. – 1 y. 9(12,5)  2-6 y. 21(16,1)  7-12 y. 85(34)  Total115(25,4)  P=<0,001  ① HAV IgG seroprevalance was higher in children of low monthly income families (36.1%, n=78; P<0.001) than in the intermediate (17%, n=31) and high income families (11.1%, n=6) | **HAV Aşısı + n (%)**  6 m. – 1 y. none  2-6 y. 32 (24,6)  7-12 y. 10 (4)  Total 42 (9.3) p=<0,001  anti-HAV IgG seropositivity in patients with no vaccination was 16.1%.  HAV vaccination rate was significantly lower in low income families (1.8%, n=4; P<0.001) than in the intermediate income (16.3%, n=30), and high income families (14.8%, n=8) | **7/9** |
| **Balamtekin et al., (2006)[36], Central anatolia region Kayseri,** | none  N:310 | This study was performed to detect seroprevalence of hepatitis A and to investigate the relationship between socioeconomical status and hepatitis A infection in the children of the military personnel living in Kayseri. | **Age**: 2-18 year  **Female:** 162  **Male:** 148  healthy military personnel child (without jaundice) | December2002 -June 2004 | ELISA (Equipar, Italy) | Anti-HAV IgG Presented according to demographic variables | - | **Anti HAV IgG+ n(%)**  **2-6 year**  12 (9.8)  **7-12 year**  30 (25.9)  **13-18 year** 49 (68.1)  male 45 (30.4)  female 46 (28.4)  **Monthly income status**  **(Turkish lira)**  >2000 YTL 15 (20.8)  1000-2000 YTL 33 (24.6)  <1000 YTL  43 (41,3) | ① The study was conducted in unvaccinated children | **7/9** |
| **Pirinççioğlu et al., (2018)[37], South eastern anatolia region Diyarbakır,** | Prospective  N:600 | This study aimed to identify anti-HAV seropositivity in children in 3 different schools in Diyarbakır, Turkey, to evaluate the risk factors influencing prevalence, and thus to develop strategies to prevent infection. | **Age mean:** 10,5 (7-14 years)  **Female:** 309  **Male:** 291 | 29.09.2011-04.10.2011 | Anti-HAV total antibodies (IgM + IgG) were analyzed by Electro Chemiluminescence immunoassay (ECLIA) using a Roche Cobas E 601 device within 2 h after samples were taken from the freezer and thawed to room temperature. A Cobas Elecsys Anti-HAV reagent kit was used for the analyses and the values were evaluated as IU/L. | **Anti HAV IgG n(%)**  247(%45,7) | - | **Anti HAV IgG n(%)**  **Male** 120 (41,2)  **Female** 154 (49,8)  **7 year**  10 (13,3)  **8 year**  18 (23,7)  **9 year**  27 (37,0)  **10 year**  33 (46,0)  **11 year**  36 (46,8)  **12 year**  44 (60,3)  **13 year**  50 (66,7)  **14 year**  56 (73,7)  We found that anti-HAV seropositivity increased as age increased and the difference was statistically significant.  x2=92.391  (p<0,001) |  | **7/9** |
| **Karaman et al.,**  **(2015)[38],** **eastern anatolia region**  **Van,** | none  N:510 | The aim of this study was to evaluate the prevalence of anti-HAV IgG in children aged from 1 to 15 years old and to determine influencing factors, such as age, demographic characteristics, socio-economic status, and housing conditions associated with the prevalence of this infection. | **Age:** 1-15 year  **Female:** 209  **Male:** 301 (%59,1)  1-4 year (n=141),  5-8 year (n=151),  9-12 year (n=146),  13-15 year (n=72)  **Socio-economic situation**  Adequate (medium/high)  n=240  Insufficient (low) n=270 | October-December 2009 | enzyme-linked immunosorbent assay (ELISA; Architect Systems ve Abbot Diagnostics Division, ABD) | **Anti-HAV IgG** %54,9 | - | **Anti HAV IgG+ n(%)**  **Gender**  Female 120 (41)  Male 160 (59)  x2 ^:^ 0,904, P=0,342  **Age**  1-4 year 37 (26,2)  5-8 year  79 (52,3)  9-12 year  106 (72,6)  13-15 year  58 (80,6)  x ^2^ : 84,799, P=0,001  **Socio-economic situation**  Adequate (medium/high)  108 (45)  Insufficient (low)  172 (63,7)  *x*^2^: 17,952, P=0,001  Multivariate regression analysis for the anti-HAV IgG (+) model (Adj OR, 95% CI, p)  Socio-economic situation  Adequate (medium/high)  0.64 (0.25-1.38), 0.09  Insufficient (low)  1.16 (0.73-1.85), 0.52 | ① The study was conducted in children who had not been vaccinated. | **5/9** |
| **Deveci et al., (2014)[39], eastern anatolia region Elazığ,** | prospective study  N:1258  n: 1010 (248 patients with anti-HAV IgM positive were excluded from the study) | This study aims to determine the seroprevalence of HAV infection among children aged 1-18 years and to observe the seroprevalence changes in Elazig in Eastern Anatolia, Turkey. | 1-18 years  **Average age of 1010 pediatric patients**10,7±5,1 year (range: 1-18 year)  Male: 558 (%55,2)  Female: 452 (%44,8) | January 2011-December 2012 | Anti-HAV IgM and anti-HAV IgG markers were tested using the ELISA method (Abbott Architect I 2000 SR). | **Anti-HAV IgG n(%)**  435 (%43,1) | - | **Anti HAV IgG+ n(%)**  **Gender**  Female:199 (%45,7)  Male: 236 (%54,3)  **Age mean±SD**  11,7±5,2 years |  | **5/9** |
| **Karadeniz et al., (2017)[40], Marmara region, İstanbul** | Retrospective study  N: 3,868 | The objective of this study is to determine the current seroprevalence of hepatitis A virus (HAV) for different age groups in Istanbul, Turkey. | 1-79 yearsı | January 2011- December 2013 | microparticle enzyme immunoassay (MPEIA) method (Architect SR i1000 and i2000, Abbott Diagnostics, Germany) | Total anti-HAV IgG antibody prevalence 64.8% | - | **HAV IgG seropositivity n(%)**  Male 1,384 (66.2)  Female 1,124 (63.3)  **Anti-HAV IgG + results according to age groups n(%)**  0-16 y. 392 (55)  17-30 y. 580 (47)  31-45 y. 853 (73.6)  >46 y. 683 (89)  ① The seroprevalence increased from 50%(972/1,944) in 0 to 30 years old to 89% in patients older than 46 years (P<0.05) |  | **5/9** |
| **Güngör, (2021)[41], Mediterrian region, Uşak** | Longitudinal retrospective study  N:5.564 | This study aimed to determine viral hepatitis status in the Uşak Province of Turkey between 2009 and 2017 | The mean age was 39.9 ± 21.8 years. | 2009 – 2017  The anti-HAV IgG test results were available between 2011 and 2017. | None | 4.111 Anti-HAV IgG antibody was detected in one person (73.89%)  **Anti-HAV IgG + n/N(%)**  **2011** 587/766 (76.63)  **2012** 415/545 (76.15)  **2013** 547/770 (71.04)  **2014** 598/794 (75.31)  **2015** 551/722 (76.32)  **2016** 625/824 (75.85)  **2017** 788/1143 (68.94) | - | **Anti-HAV IgG + results according to age groups n/N(%)**  **0-5 age** 110/141 (78.01)  **6-14 age** 31/221 (14.03)  **15-24 age** 168/537 (31.28)  **25-34 age** 219/311 (70.42)  **35-44 age** 281/296 (94.93)  **45-54 age** 308/309 (99.68**)**  **55-64 age** 264/264 (100)  **65+ age** 470/470 (100)  ① Anti-HAV IgG was detected more frequently in women than in men (76.26% and 70.06% respectively, p<0.001). There was a significant decrease in the anti-HAV IgG seropositivity rate between 2011 and 2017. |  | **5/9** |
| **Şirin et al., (2022)[42], Marmara region İstanbul** | Retrospective study  N: 10132 | The aim of this study was to examine the change in HepA seroprevalence and identify the population susceptible to HepA. | Age (year), median (IQR), [min-max]  44 (35-54) [18-98] | January 2016 -January 2019 | chemiluminescence microparticle immunoassay (Architect i2000, Abbott, U.S.) | Anti-HAV IgG Positivity  %60,1 (n=6088) | - | Anti-HAV IgG Positivity n(%60,1) (n=6088)  Male 3108 (66.6)  Female 2980 (54.5)  18-24 years 944 (29.0)  25-29 years 837 (49.7)  30-34 years 689 (60.6)  35-39 years 784 (76.6)  ≥40 years 2834 (93.3)  Healthcare Professionals 2302 (62.6) |  | **6/9** |
| **Köse et al., (2013)[43], aegean region İzmir** | cross-sectional study  N:2156 | This study was aimed to determine the population-based seroprevalence of HAV in Izmir. | mean age was 44.05±16.83 (range: 15-94) years.  Female:1338(%61,9)  Male: 820(%38,1)  **15-19 y**. 135(%6,4)  **20-24 y.** 144(%8.8)  25-29y. 197(%9.3)  30-34 y. 201(%9.8)  35-39 222(%10,5)  40 y.+ 1208(%57,4)  <1000 TL. 1297 (64.2)  1000-2000 TL. 518 (25.6)  >2000 TL. 206 (10.2) | January 2010 -March 2010 | Enzyme linked immunosorbent assay (ELISA) (Diasorin, Italy) | Anti-HAV IgG positivity was % 93.9 | - | **Anti-HAV IgG Positivity n(%)**  **Female:**1239(93,7)  **Male:7**68(94.1)  **15-19 y.** 92(68,1)  **20-24 y**. 108(73,6)  **25-29 y.** 175(88.8)  **30-34 y.** 191(94.6)  **35-39 y**. 216(97,3)  **40y.+** 1197(99.1)  <1000 TL. 1233 (95.1)  1000-2000 TL. 471 (90.9)  >2000 TL. 194 (94,2) |  | **8/9** |
| **Arabacı et al. (2009) [44], Marmara Region, Çanakkale** | Retrospective study  **N:** 1363 | The aim of this study is to determine Hepatitis A seroprevalance and its distribution in different age groups, and also to determine HAV incidence among acute hepatitis cases in the Canakkale province of Turkey. | 1.group  mean age was 31.97±18.61 (range: 0-91) years.  Female: %57.1  Male: %42.9 | January 2006-December 2007 | It was studied using Bio-rad Access HAV IgG and IgM kits on the Beckman-Coulter Access2 device. | **HAV IgG**  %78.87 (1075/1363) | - | **Distribution of HAV IgG and seropositivity by age groups**  **Poz./total (%)**  0-6 y**.** 38/77 (49.3)  7-11 y. 71/131 (54.2)  12-16 y. 46/76 (60.5)  17-21 y. 118/192(61.4)  22-26 y. 98/139 (70.5)  27-31 y. 99/114 (86.8)  32-36 y. 114/123(92.7)  37-41 y. 87/95 (91.5)  42-46 y. 111/113(98.2)  47-51 y. 84/86 (97.6)  >52 y. 209/217 (96.3) |  | **6/9** |
| **Ungan et al., (2002)[45], Central Anatolia Region Ankara** | -  N:137  **n:114**  Blood could not be taken from 18 children because their parents did not give permission. Vaccinated (5) children were excluded from the study when calculating the prevalence. | Our major aim was to access the seroprevalence of anti-HAV IgG antibodies in the 4–6-year-age group and also the rate of immunized children. | 4-6 year  Mean±SD 5.184 ± 0.771 yıl | 1 May and 1 July 2000. | The anti-HAV IgG was assessed from blood samples using a commercially available test for total anti- HAV | Among all children (n = 114), (n = 13) were considered seropositive (11.4%). | - | **none** | ① It was determined that five out of 137 children (3.65%) had received the HAV vaccine at least once. | **8/9** |
| **Okur et al., (2011)[46], eastern anatolia region, Van** | Retrospective  N:3409 | The aim of this study is to determine Hepatitis A virus (HAV) seropositivity in Van province and around. | 0-18 year  Mean±SD  7 ± 4.3 years  Female: 1516’sı (% 44.5)  Male: 1893’ü (% 55.5) | January2007-December 2008 | It was studied with the MEIA (Microparticle Enzyme Immune Assay) method with the Abbott AxSYM (USA) device and kits. | **Anti-HAV IgG**  % 69.9 (2384/3409) | - | **Anti-HAV IgG Positivity n(%)**  0-2 y. 291(53,1)  3-5 y. 448 (55,7)  6-10 y. 1000(74,4)  11-15 y. 502(90)  15-18 y. 143(92,3)  Female %72,6  Male %67,7 |  | **6/9** |
| **Kalfaoğlu et al., (2017)[47], aegean region Izmir** | Cross-sectional  N: 424 | The aim of the study is to determine the seroprevalence of HAV and HEV infection and its associated factors in Izmir city center. | **Age:** 7-80 | November 2013-January 2014 | ELISA | **HAV IgG:**  83.2% | - | **Gender: % (n)**  -Female: 85.2 (207)  -Male: 83.4 (151)  **Age: % (n)**  - 7-14 years: 18.2(2)  - 15-19 years: 42.9 (6)  - 20-29 years: 55.0 (33)  - 30-39 years: 87.5 (98)  - 40-64 years: 96.3 (208)  - ≥ 65 years 100.0 (11)  ① HAV IgG seropositivity was found to be significantly lower (p: 0.000) in the pediatric age group. Again, there was a direct proportion between age and HAV IgG positivity, and the anti-HAV prevalence peaked at the ages of 14-19.. |  | **8/8** |
| **Uzun Karaayak et al., (2013)[48], aegean region Izmir** | Retrospective  N:4157  n: Anti-HAV IgG and Anti-HAV IgM from 3,887 patients  tests were requested. | The aim of the study is to investigate the frequency of Hepatitis A and E transmitted through fecal-oral route in the adult population admitted to the hospital. | **Age:** >18 | January-December 2012 | Advia Centaur XP Bayer-Siemens (Germany) | **Anti-HAV IgG:** 85.2% | - | **Anti-**  **HAV IgG**  -Female: 48.9%  -Male: 51.1% |  | **6/9** |
| **Ünver et al., (2014)[49], Eastern anatolia region, Muş** | Retrospective  N:1432  n=369; 0-14 years old  n=1063; ≥14 | The aim of the study is to determine the HAV incidence according to age in patients admitted to Muş State Hospital and to detect HAV seropositivity in this region. | Children 0-14 years old and adults ≥14 years old | 01 January - 31 October 2014 | ELISA (Architect-Abbott Wiesbaden,Almanya) | **Anti-HAV IgG:** 95.86%  (Adult age group) | - | **Anti HAV IgG**  **Age**  0-5 years: 18.9 %  6-10 years: 29.7 %  11-14 years: 51.3 %    15-20 years: 92 %  21-30 years: 93.8 %  31-40 years: 98.5%  41-50 years: 100%  51-60 years: 100 %  >60 years: 100 % |  | **7/9** |
| **Şimşek-Bozok ve Bozok, (2021)[50], Central anatolia region Niğde,** | Retrospective  n: 2565 | The aim of the study is to investigate the seroprevalence of hepatitis A, B and C in Niğde province and compare it according to age groups and gender. | All age groups | November 2016-November 2019 | ELISA | **Anti-HAV IgG**:  63.8% | - | **Age**  0-23 month: 55.6%  2-6 years: 74.2%  7-10 years: 69.1%  11-19 years: 22.6%  20-29 years: 43.7%  30-39 years: 85.3%  40-49 years: 96.5%  50-59 years: 99.3 %  60-69 years: 100  70 yaş ve üzeri: 99.3  **P: <0.001**  <20: 38.5%  ≥20: 74.3%  -Anti-HAV IgG positivity in patients aged 20 and over (74.3%) was significantly higher than in patients under 20 years of age (38.5%) (p< 0.001).  -When the group under 20 years of age is evaluated for anti-HAV IgG positivity; Anti-HAV IgG positivity of patients in the 0-7 age group (70%) was significantly higher than that of patients in the 7-19 age group (37.7%) (p< 0.001).  **Gender:** Female: 60.7%  Male: 67.3%  - Anti-HAV IgG positivity was significantly higher in men than in women in all patients included in the study (p < 0.001).  -Anti-HAV IgG positivity was significantly more common in men (p< 0.001) in patients aged 20 and over, while no gender difference was detected in patients under 20 years of age (p: 0.355). |  | **6/9** |
| **Çiçek- Çopur et al., (2012)[51], Black Sea region, Rize** | Retrospective  N: 1173 | The aim of the study is to determine the hepatitis A, B and C seropositivity rate in pediatric patients in Rize province, which has not been investigated so far, to compare it with other regions of the country and to contribute to the limited number of epidemiological studies in pediatric patients.. | **Age (mean):** 5.61±4.21 years  **Gender (n, %)**  Female: 425, 36.2%  Male: 748, 63.8% | 01.01.2010-31.12.2011 | ELISA | **Anti-HAV IgG:**  29.5 % | - | **Anti-HAV IgG**  **Gender**  Female: 47%  Male: 53%    The difference in terms of gender is statistically significant  was found (p<0.0001). |  | **7/9** |
| **Ertürk et al., (2013)[52], Black Sea region Rize,** | Retrospective  N: 1112 | The aim of the study is to determine the seropositivity of hepatitis A in patient groups of different age groups admitted to the hospital and the rate of hepatitis A in cases with acute hepatitis, to compare the rates with the rates reported in the same age group in different regions of the country, and to use the hepatitis A vaccine recently as the Expanded National Immunization Program was initiated to determine the hepatitis A seropositivity rate in cases with acute hepatitis. to determine based data. | **Age (mean):** 33,5±13,5  **Gender (n, %)**  Female: 486, 43.7%  Male: 626, 56.3% | 01.01.2011- 31.12.2011 | ELISA | **Anti-HAV**  **IgG:** 75% | - | **Anti-HAV**  **IgG**  **Gender**  -Female: 43%  -Male: 57%  p:0.647.  - Prevalence in the study; While it was 47.3% in the 17-27 age group, it was 92% in the over 50 age group. Anti-HAV IgG seropositivity in the adult age group; young adulthood period between the 1st age group (17-27) and the other middle [2. age (28-39) and 3rd age (40-50)] groups and late adulthood [4th age]. A significant difference was detected between the periods of age (51-60) and 5th age (61-70)]. All these data reveal that HAV seropositivity increases proportionally as age increases. |  | **7/9** |
| **Kural, (2019)[53], Marmara Region, Istanbul** | Cross-sectional  N: 546  School 1 (low-middle socioeconomic level);  n: 284  School 2 (high socioeconomic level);  n: 262 | The aim of the study is to compare hepatitis A and hepatitis B vaccination rates and seroprevalences in two schools with different socioeconomic levels located in the same environment. | **School 1**  **Age (mean):**  10.7±2.2  **Gender (n)**  Female: 159  Male: 125  **School 2**  **Age (mean):**  9.8±2.4  **Gender (n)**  Female: 141  Male: 121 | 2003 | Serological study was performed using BioRat Anti-HAV IgG (Pasteur®) kits. | **Anti HAV IgG:** 23.4% | - | School 1: 11.3%  School 2: 36.6%  p<0.001* | **Vaccinated** School 1: 1.8%  School 2: 29.8%  p<0.001* | **6/8** |
| **Gündem and Ataş, (2022)[54], Central anatolia region Konya** | Retrospektive  N: 760 | The aim of the study is to determine HAV seropositivity in pediatric patients in Konya, to investigate its distribution according to age groups, gender and seasons, and to compare the obtained data with those reported in previous studies. | **Age (mean)** :10.5±5,1 years  **Gender, n(%)**  Female: 307 (40.4)  Male: 453 (59.6) | January 2019-April 2020 | ELISA | **Anti-HAV IgG**: 52.2% | - | **Age groups**  **Anti-HAV IgG**  0-2 age: 5.8%  3-8 age: 43.8%  9-14 age: 21.9%  15-18 age: 28.5%  The highest anti-HAV IgG seropositivity rates were found in the 3-8 age group and were significant compared to other age groups (p<0.05).  **Gender**  **Anti-HAV IgG**  Female: 35.2%  Male: 64.8%  p:0.017 |  | **7/9** |
| **Köroğlu et al., (2014)[55], Marmara Region, Sakarya** | Retrospektive  N:2003 | The aim of the study is to investigate the seroprevalence of Hepatitis A virus (HAV) infection for all age groups in Sakarya province and to compile previously published data from our country. | **Age:** 0-92 | 01.01.2012-31.12.2013. | ELISA | **IgG anti-HAV**: 74.7% | - | **Gender**  **IgG anti-HAV**  Female: 39% Male: 35.7%  **Age Group**  **IgG anti-HAV**  0-10: 29.7%  11-20: 43.4%  21-30: 57.1%  31-40: 84.8%  41-50: 96.8%  51-60: 99%  61-92: 99.3%  - IgG anti HAV seropositivity in the 0-10, 11-20 and 21-30 age groups was significantly lower than the 31-40, 41-50, 51-60 and 61-92 age groups (p<0.001). |  | **7/9** |
| **Düzenli et al., (2021)[56], Central anatolia region Çorum** | Retrospective  N: 18817 | The aim of the study is to investigate the seroprevalence of hepatitis A in different age groups in Çorum, which is the reference center in the Northern part of Central Anatolia in Turkey, and to make recommendations on hepatitis A vaccination according to these age groups. | Children and adults | January 2017- January 2020 | ELISA | **Anti-HAV IgG**: 84.4% | - | **Gender**  **Anti-HAV IgG**  Female: 83%  Male: 85.8%  **Age group**  **Anti-HAV IgG**  0 – 10 years: 84.6%  11 – 20 years: 71.6%  21 – 30 years: 75.8%  31 – 40 years: 91.1%  41 – 50 years: 97.2%  51 – 60 years: 95.1%  61 – 70 years: 92.3%  71 – 80 years: 95.8%  > 80 years: 95.6%  p<0.001* |  | **7/9** |
| **Parlak et al., (2015)[57], eastern anatolia region Van,** | Retrospektive  N: 6697  n:5363 (anti-HAV IgG studied group) | The aim of the study is to determine the seroprevalence of Hepatitis A in pediatric and adult patients admitted to a training and research hospital. | Children and adults | June 2012-July 2013 | ELISA | **HAV IgG**: 89.9% | - | **Anti-HAV IgG**  Pediatric age group  0-1: 34%  2-3: 27.7%  4-5: 47.4%  6-7: 62%  8-9: 64.7%  10-11: 72:  12-13: 82.3%  Adult age group  14-19: 76.9%  20-29: 88.5%  30< : 98.3% |  | **5/9** |
| **Demir ve Turan, (2015)[58], Central anatolia region Kırşehir** | Retrospective  n: 29081  n:33012 (analyzed for anti-HAV IgG) | The aim of the study is to determine the seroprevalence of hepatitis A in patients from different age groups who applied to our training and research hospital. | **Age:**: 3-97 years | January 2009-June  2013 | ELISA | **Anti-HAV IgG**: 87.3%  - When the seasonal distribution of acute HAV cases was evaluated, it was determined that the infection was most common in August (11.3%), September (13.6%) and December (13.6%), and was least common in February (2.3%). | - | **Age groups**  **Anti-HAV IgG**  0-5: 99.7%  6-10: 91.2%  11-20: 61.2%  21-30: 61.4%  31-40: 91.9%  41-50: 97%  51-60: 97.8%  61-70: 98.6%  71 years and over: 98.1% |  | **7/9** |
| **Yildiz ve Narsat, (2022)[59], Black Sea Region, Kastamonu** | Retrospective  N: 19885 | The aim of the study is to determine the seroprevalence of hepatitis A, B and C in children in Kastamonu province, to evaluate whether there is a change in prevalence according to years and patient age, and to review whether there is a change in the seroprevalence of hepatitis A virus with hepatitis A vaccine. | **Age (mean):** 10.0±5.0 (1-17 years).  **Gender**  Female: 38.5%  Male: 61.5% | 01.01.2012-21.12.2021 | ELISA | **Anti-HAV IgG:** 29.3%  **Years**  **2012**  Anti-HAV IgG: 25.4%  **2013**  Anti-HAV IgG: 23.9%  **2014**  Anti-HAV IgG: 24.3%  **2015**  Anti-HAV IgG: 34.3%  **2016**  Anti-HAV IgG: 20.2%  **2017**  Anti-HAV IgG: 36.3%  **2018**  Anti-HAV IgG: 43.3%  **2019**  Anti-HAV IgG: 37.1%  **2020**  Anti-HAV IgG: 51.6%  **2021**  Anti-HAV IgG: 50.7% | **-** | **-** | Unvaccinated: 22.6%  Vaccinated: 98.8% | **7/9** |
| **Yentür-Doni et al., (2017)[60], south eastern anatolia region Adiyaman, Batman, Diyarbakır, Gaziantep,**  **Kilis, Mardin, Siirt, Şanlıurfa, and Şırnak,** | Cross-sectional  N:705 | The aim of the study is to determine the antihepatitis A virus (HAV) seroprevalence, risk factors and knowledge of female agricultural workers living in the Southeastern Anatolia Region of Turkey. | **Age (mean):** 36.16 ± 9.79  **Gender**  Female: 100% | January 2013 - December 2013  (Learned by sending an e-mail to the author) | ELISA | **Anti-HAV IgG**: 99.1% | **-** | **Age groups**  Adolescent (15–19 years old): 90.9%  Adults (20–49 years old): 99.3%  **Poverty**  Yes:99.2%  No:98.5% |  | **8/8** |
| **Çalık et al., (2019)[61], Aegean region,**  **İzmir** | Cross-sectional seroprevalence survey  N=1336 | This study aimed to investigate the seroprevalence of hepatitis A in different age groups in a region with low and medium socioeconomic levels in Izmir province, with a prospective field study. | A total of 1336 individuals participated in the study, 1000 (74.9%) of whom were women and 336 (25.1%) were men. 426 people (31.9%) were aged 16 and under and a survey was not administered to these people.  Of the 910 people, 676 (74.3%) are women and 234 (25.7%) are men.  Of the 910 participants, 46 (5.1%) were illiterate, 48 (5.3%) were literate, 423 (46.5%) were primary school graduates, 120 (13.2%) were secondary school graduates, 199 (21.9%) were high school graduates and 74 (8.1%) were university graduates.  851 of 910 people answered the questions about socioeconomic level completely, but the data of 59 people were not evaluated in this regard due to incomplete answers.  Of the participants who fully answered the questions about their socioeconomic level and were evaluated, 280 (32.9%) were found to be low, 441 (51.8%) were medium, and 130 (15.3%) were high. | 10 September 2015-1 September 2016 | Enzim immünoessey yöntemiyle (Dia.Pro  Diagnostic BioProbes Srl, Milan, İtalya), ETI-Max (DiaSorin,  The presence of anti-HAV IgG was investigated using the device (Vercelli, Italy). | **Anti HAV IgG (+) n, (%):**  989 (%74)  **Anti HAV IgG (+) n(%) by socioeconomic level**  **Low=** 245 (87.5)  **Medium=** 344 (78)  **High=** 103 (79.2) | - | Hepatitis A seropositivity was detected in 81.8% of individuals (n=851) who fully answered questions about socioeconomic level. A statistical relationship was found between socioeconomic level and hepatitis A seropositivity status (p=0.005). | ① Vaccinated and unvaccinated individuals. | **8/9** |
| **Bedge et al., (2015)[62], Central Anatolia Region**  **Ankara** | Cross-sectional seroprevalence survey  N= 317 | Aimed to evaluate the persistence of naturally  acquired maternal antibodies against HAV and varicella-zoster (VZV) in a  group of healthy children between 6 and 24 months of age,  and to determine the optimal time of vaccination before  including these vaccines in a national vaccination schedule.  Furthermore, we examined the possible relations between  sociodemographic characteristics and maternal antibody  status. | The study population consisted of healthy infants who  were admitted to the Division of Social Pediatrics for 6-,  12-, 18-, and 24-month follow-up visits.  The mean age of the entire study population was 13.73  ±6.60 months (median 12, range 5.69–25.40 months). | - | ELISA | The total seropositivity was 36.9 % in the whole study population  (n=117).   Seropositivity rates were found 71 % (n=71)  in group 1, 41.4 % (n=41) in group 2, 0 % in group 3, and  8.5 % (n=5) in group 4. | - | No statistically significant differences were found in the seroprevalence  rates of HAV and VZV antibodies in terms of the  genders of children and the sociodemographic characteristics  of the families (p>0.05 in all comparisons). |  | **8/9** |
| **Görgel-Kahraman et al., (2019)[63], Aegean region**  **Manisa** | Cross-sectional seroprevalence survey  N=1223 | The aim of the study was to determine the hepatitis A seronegativity rate in the population over the age of two in Manisa province in 2014 and to examine its relationship with social determinants. | 1223 participants over 2 years old,  **Age n (%)**  **2-9 age=** 135 (11,0)  **10-19 age=** 187 (15,3)  **20-29 age=** 166 (13,6)  **30-39 age=** 183 (15,0)  **40- 49 age=** 199 (16,3)  **50-59 age=** 152 (12,4)  **60-69 age=** 107 (8,7)  **70-79 age=** 72 (5,9)  **80 years and above =** 22 (1.8)  52.1% of the research group are women, 77.2% live in surrounding districts, 25.8% are outside the workforce, 18.3% are production workers, and 15.4% are agricultural workers. | 18.03.2014–22.06.2014 | Anti-HAV positivity was studied by the electrochemiluminescence method with the Cobas e 411 (Roche Diagnostics GmbH, Mannheim, Germany) analyzer and the anti-HAV kit (Roche Cobas Elecsys) compatible with this analyzer. | **Distribution of HAV negative people by age groups (%)**  **2-9 years =** 78.5  **10-19 years=** 65.8  **20-29 years=** 31.3  **30-39 years =** 5.5  **40-49 years =** 2.5  **50-59 years =** 0.7  **60-69 years =** 0.9  **70-79 years =** 0  **80 years and above =** 4.5 | A significant relationship was found between hepatitis A seronegativity and annual per capita equivalent income. When adjusted for age, it was determined that having an equivalent annual per capita income of ≤3265 TL had a reducing effect on hepatitis A seronegativity (OR: 0.61, 95%CI: 0.42-0.90). | In the study, hepatitis A seronegativity was found to be 24.4%. Hepatitis A seronegativity is highest in the 2-9 age group, corresponding to primary school and below. Seronegativity decreases with increasing age, varying between 0.0-5.5% in the 30-year age group. | ①Since the routine hepatitis A immunization program in our country started in 2012 and the samples were collected from two-year-olds in 2014, the research results reflect the pre-vaccination period. | **9/9** |
| **Vançelik and Güraksin, (2006)[64], eastern anatolia region**  **Erzurum** | Cross-sectional seroprevalence survey  N=392 | The aim of the study was to investigate HAV seroprevalence and association with socio- demographic variables. | **Age:** 0-29 yaş  **Gender (n, (%))**  **Female:** 193 (49.2)  **Male:** 199 (50.8)  **Age n(%)**  **0=** 13(3.3)  **1-4=** 50 (12.8)  **5-9=** 74 (18.9)  **10-14=** 89 (22.7)  **15-19=** 60 (15.3)  **20-24=** 48 (12.2)  **25-29=** 58 (14.8) | 2002 | All the were tested by assay technique (Behring anti- HAV OQEC 11) for the of hepatitis A Ig G (Ig G) | **HAV (+) (n, (%))**  330 (84.2) | **%95**  **Age**  P-valuve=0.001  Odd Ratio=1.0  Lower Border= 1.0  Upper Border=1.1 | There was relation between age and of HAV (χ2: 23.8, p<0,001).  **HAV (+) Age n(%)**  **0=** 10 (76.9)  **1-4=** 33 (66.0)  **5-9=** 57 (77.0)  **10-14=** 83 (93.3)  **15-19=** 54 (90.0)  **20-24=** 42 (87.5)  **25-29=** 51 (87.9)  The results of logistic regression analysis displayed that there is relation between anti-HAV existence and age presence of toilet in house and number of persons in the family. It has, however, no relation with sex, history of jaundice, settlement localisation, tap water and structure of house. |  | **8/8** |
| **Gülgün et al. (2014)[65], central anatolia region**  **Kayseri** | Retrospective study  N= 1032 | This study was conducted to detect anti-HAV IgG seropositivity in young adults admitted to a public hospital in Kayseri. | **Age:** 16-24 yaş  **Gender (n, (%))**  **Female:** 263 (% 25.4)  **Male:** 769 (74.9%)  **The average age:**  Female: 22.3± 2,1 Male: 20.6±3.9. yıl | 01 January 2006–01 April 2010 | Anti-HAV IgG (Access2, Beckman Coulter-USA, chemoluminescence method) | **HAV (+) n(%)**  **Female:** 150 (%57.0)  **Male:** 488 (%63.4) | - | There was no statistically significant difference in terms of gender and age (p>0.05). |  | **7/9** |
| **Özen et al. (2006)[66], eastern anatolia region**  **Malatya** | Descriptive study  N: 685 | The aim of this study was primarily to determine the anti-HAV IgG seropositivity in cases admitted to the Department of Child Health and Diseases of a university hospital serving in Malatya and to determine whether the frequency of HAV infection has decreased similar to that in developed countries and provinces of Turkey with higher socioeconomic values. | **Age:** 2-16  **Gender (n, (%))**  **Female:** 324 (%47,3  **Male:** 361 (%52,7) | January 2004 - July 2005 | Anti-HAV immunoglobulin M (IgM) and IgG variants (Dade-Behring) were examined by ELISA method. | **Number of Cases n(%),**  **HAV IgG (+) n(%)**  178 (25,9)  **Age**  **3-6:** 286 (41,7) 50 (17,5)  **7-16:** 399 (58,3) 128 (32,1)  χ2: 18,46  **Gender**  **Female:** 324 (47,3) 90 (27,7)  **Male:** 361 (52,7) 88 (24,3)  p:0,000017  **City**  **Malatya:** 564 (82,4) 149 (26,6)  **Outside Malatya:** 121 (17,6) 29 (23,9)  χ2: 0,31  p: 0,576  **Residential area**  **Town center:**  570 (83,2) 152 (26,6)  **Rural**: 115 (16,8) 26 (22,6)  χ2: 0,82  p: 0,365 | - | It was found that the seropositivity rate was higher in school-age children and was statistically significant (χ2: 18.46, p<0.00001). | no vaccination | **7/9** |
| **Atabek et al. (2004)[67], central anatolia region**  **Konya** | Cross-sectional seroprevalence survey  N=210 | The present study was undertaken to determine the  seroprevalence of antibodies to HAV and HEV and to  help assess the value of anti-HAV vaccination. | **Rural areas**  n: 100  **Age:**1-18 age (9,37±7,3)**Gender (n)**  **Female:** 51  **Male:** 49  **Urban areas**  n: 110  **Age:**1-18 age (8,2±6,25)**Gender (n)**  **Female:** 52  **Male:** 58 | 2001-2002 | anti-HAV total (anti-HAV IgM ve anti-HAV IgG)  enzyme linked immunoassay | **Anti-HAV (+) (n, (%))**  147 (70)  **Rural areas**  **1-6 age**: 19 (67.8)  **7-12 age**: 32 (91.4)  **13-18 age:** 36 (97.2)  **Total:** 87 (87)  **Urban areas**  **1-6 age**: 8 (25.7)  **7-12 age**: 15 (39.4)  **13-18 age:** 37 (90.8)  **Total:** 60 (54.4) | - | **Anti-HAV (+) (n, (%))**  **Rural areas**  87, (87)  **Economical condition**  Poor, n (%): 65(74.7)  Adequate, n (%): 22(25.2)  **Urban areas**  No tested: 60  **Economical condition**  Poor, n (%): 43(71.6)  Adequate, n (%): 17(28.3) | No vaccination | **8/8** |
| **Karaşahin and Karaşahin, (2023)[68]**  **Erzurum**  **Eastern Anatolia region of Turkey** | Retrospectice - Cross-sectional seroprevalence survey  N= 25.884 | The aim of this study was to  evaluate the prevalence of HAV seropositivity  after the inclusion of the HAV vaccine in the  national vaccination program and to determine  demographic risk factors for susceptibility in the  pre-vaccination population. | **Age:** 1-100 years ( 32.0)  **Gender (%)**  **Female:** 45.5  **Male:** 54.5  **Age groups (median age for each group, (age range)):** A=4 (1-8), B=7 (4-13), C=12 (6-19), D=17 (10-25), E=24 (16-32) , F=30 (23-38) and G=53 (29-100). | Between  2008 and 2019 | Anti-HAV IgG antibody quantification was performed by enzyme-linked immunosorbent assay in the hospital microbiology laboratory. | **Anti-HAV (+) (n, (%))**  21,124, (81.6%) | - | HAV seropositivity among people born in 2012 or later was lowest (43.8%) in those born in the Southeast Anatolia region and it was over 60% in other regions.  Anatolia regions had higher seropositivity than  those in other regions. The rate of anti-HAV positivity  increased with age in all regions and was  over 85% among people born in 1981 or earlier.  Total Anti-HAV IgG positivity differed significantly in all pairwise comparisons between age groups (p<0.001) except between those born in or after 2012 and those born in 1988-1993 (p=0.204).  The lowest rate of anti-HAV IgG positivity was in people born in 2006-2011 (43.5%) and the highest was in those born before 1981 (97.8%) (p<0.001).  Anti-HAV IgG positivity was significantly higher in males (82.8%; n=11,679) than in females (80.2%; n=9,445) (p<0.001).  This difference was due to the higher rate of anti-HAV IgG positivity in males born between 1982 and 1999 (p<0.001). Anti-HAV IgG positivity was significantly higher among rural dwellers (89.2%; n=12,435) than urban dwellers (72.8%; n=8,689) (p<0.001). This difference was associated with the higher HAV seropositivity in rural dwellers born between 1982 and 2005 (p<0.001). |  | **8/8** |
| **Köse ve ark., (2017)[69],** **aegean region, İzmir** | Descriptive study  N: 2003 pregnant women | In this study, hepatitis B surface antigen (HBsAg), hepatitis B surface antibody (antiHBs), hepatitis C antibody (anti-HCV) were collected from pregnant women who applied to the Pregnancy Clinic of Izmir Aegean Maternity and Gynecology Training and Research Hospital and were referred to the Blood Collection Center. ) and hepatitis A virus antibody (anti-HAV IgG) results were examined to investigate their seroprevalence. | **Age**: 27±3 (17-44)  **Job:**  1797 (89.7%) were housewives, 108 (5.4%) were workers, 72 (3.6%) were self-employed, 22 (1.1%) were civil servants and 4 were (0.2%) are students. | 01 December 2010-30 September 2011 | ELİSA | **Anti HAV IgG (+) (n, %)**  1767 (% 88,2)  **17-26 Age:** 763 (%43,1)  **27-36 Age:** 942 (%53,3)  **37-44 Age:** 62 (%3,5) | **-** | - | **no vaccination** | **7/9** |
| **Afyon ve ark., (2018)[70], All regions in Turkey** | Descriptive, Retrospectice Study  N: 925  **Student n (%):** 802 (%86.7)  **Personnel n (%):** 123 (%13.3)  **Geographic distribution by place of birth for all participants (n, (%))**  **Marmara:** 322 (34.8)  **Aegean:** 127 (13.7)  **Mediterranean:** 43 (4.6)  **Central Anatolia:** 169 (18.3 )  **Black Sea:** 140 (15.1)  **Southeastern Anatolia:** 42 (4.5)  **Eastern Anatolia:** 82 (8.9)  **Total:** 925 (100) | Determination of the rate of HAV seronegativity and the need for immunization in Naval Academy students and Naval Academy personnel. | **Student**  **Age:** 20-24 (21(0))  **Gender (n, (%))**  **Female:** 21 (%2.6)  **Male:** 781 (97.4)  **Geographic distribution by place of birth (n, (%))**  **Marmara:** 274 (34.2)  **Aegean:** 120 (15.0)  **Mediterranean:** 39 (4.9)  **Central Anatolia:** 149 (18.6)  **Black Sea:** 108 (13.5)  **Southeastern Anatolia:** 41 (5.1)  **Eastern Anatolia:** 71 (8.9)  **Personnel**  **Age:** 24- 56 (40.01±7.20)  **Gender (n, (%))**  **Female:** 8 (%6.5)  **Male:** 115 (86.7)  **Geographic distribution by place of birth (n, (%))**  **Marmara:** 48 (39.0)  **Aegean:** 7 (5.7)  **Mediterranean:** 4 (3.3)  **Central Anatolia:** 20 (16.3)  **Black Sea:** 32 (26.0)  **Southeastern Anatolia:** 1 (0.8)  **Eastern Anatolia:** 11 (8.9) | 2013-2016 | EIA (Enzim İmmunassay) | **Anti HAV IgG (+) (n, %)**  **All Participants**  310 (33.5)  **Marmara:** 96 (29.8)  **Aegean:** 7 (21.3)  **Mediterranean:** 13 (30.2)  **Central Anatolia:** 57 (33.7)  **Black Sea:** 45 (32.1)  **Southeastern Anatolia:** 28 (77.7)  **Eastern Anatolia:** 44 (53.7)  **Total:** 310 (33.5)  **Student**  **Marmara:** 54 (19.7)  **Aegean:** 23 (19.2)  **Mediterranean:** 10 (25.6)  **Central Anatolia:** 41 (27.5)  **Black Sea:** 17 (15.7)  **Southeastern Anatolia:** 27 (65.9)  **Eastern Anatolia:** 36 (50.7)  **Total:** 208 (25.9)  **Personnel**  **Marmara:** 42 (87.5)  **Aegean:** 4 (57.1)  **Mediterranean:** 3 (75.0)  **Central Anatolia:** 16 (80.0)  **Black Sea:** 28 (87.5)  **Southeastern Anatolia:** 1 (100)  **Eastern Anatolia:** 8 (85.7)  **Total:** 102 (82.9) | **-** | Seropositivity rates showed a statistically significant difference (p<0.001) between student and staff groups.  The personnel group was divided into two: officers and petty officers as a group (n=69, 56.1%) and civilian civil servants as a separate group (n=54, 43.9%). The HAV seronegativity rate was significantly (p=0.042) higher (23.2% vs. 9.3%).  According to geographical regions, the HAV seronegativity rates in the Southeastern Anatolia and Eastern Anatolia regions were significantly lower (33.3% and 46.3% in all participants, 34.1% and 49.3% in students, respectively) and the HAV seronegativity rates showed a significant difference (p<0.001) between the regions.  Within the personnel group, the age variable between those born in the Southeastern Anatolia and Eastern Anatolia regions (n=12) and those born in other geographical regions (n=111) (42.33±7.29 years and 28-56 years, 39.76±7.18 years and 24-56 years) No significant difference (p=0.241 and p=0.429, respectively) was detected in terms of range) and HAV seronegativity rates (25% and 16.2%).  When only the student group is included, the age variable between the Southeastern Anatolia and Eastern Anatolia region group (n=112) and the other regions group (n=690) [21(1) years and 20-24 years and 21(0) years and 20 years) -24 years range, p=0.020] and HAV seronegativity (43.8% to 79%, p<0.001), while there was no significant difference in terms of female gender distribution (1.8% to 2.8%, p=0.755). |  | **7/9** |
| **Ağca ve Toklu, (2013)[71], Aegean region, Uşak** | Retrospective study  N: 1260  Uşak Public Hospital  n: 799,  Tavsşanlı Public Hospital  n: 461 | In this study we aimed to determine the seroprevalance of HAV se- ropositivity in two different hospitals in the Aegean region of Turkey. | **Uşak Public Hospital**  **Age:**  27,7 ( between 5 months and 86 years of age)  **Age group (n)**  **0-5:** 53  **6-10:** 44  **11-20:** 116  **21-30:** 334  **31-40:** 142  **41-50:** 39  **51-60:** 38  **>61:** 33  **Gender n, (%)**  **Female:** 625 (78 %)  **Male:** 174 (22 %)  **Tavsşanlı Public Hospital**  **Age:** 38,0 ( between 1 month and 95 years of age)  **Age group**  **0-5:** 12  **6-10:** 14  **11-20:** 54  **21-30:** 89  **31-40:** 90  **41-50:** 81  **51-60:** 73  **>61:** 48  **Female: .** 247 ( 54 %)  **Male:**  214 (46 %) | January 2011 to December 2011 | chemiluminescence microparticle immunoassay (CMIA) | **Anti HAV IgG (+) (n, %)**  **Uşak Public Hospital**  **0-5:** 9 (16.9)  **6-10:** 10 (22.7)  **11-20:** 65 (56.0)  **21-30:** 278 (83.2)  **31-40:** 137 (96.4)  **41-50:** 36 (93.3)  **51-60:** 36 (94.7)  **>61:** 33 (100)  **Total n,(%):** 604 (75.5)  **Tavsşanlı Public Hospital**  **0-5:** 3 (25)  **6-10:** 2 (14.2)  **11-20:** 26 (8.1)  **21-30:** 69 (77.5)  **31-40:** 82 (91.1)  **41-50:** 77 (90.5)  **51-60:** 67 (91.7)  **>61:** 45 (93.7)  **Total n,(%):** 371 (80.4) | **-** | There was significant difference between age groups in both of the hospitals; Anti HAV Ig G seropositivity in 0-10 years of age group was significantly lower than that of the group older than 10 years group (p<0,05). There was no statistically significance between the age groups of Tavsanli and Usak State Hospitals |  | **7/9** |
| **Cesur ve ark., (2002)[72], Central Anatolia Region, Ankara** | Descriptive study  N: 1046 | The aim of this study is to investigate the seroprevalence of Anti HAV IgG and Anti HEV total antibodies in the adult age group in the Ankara region. | **Age:** 15-75 (mean: 32.3)  **15-30:** 294  **30-45:** 456  **45-60:** 242  **>60:** 54 | September 2000- July 2001 | ELİSA | **Anti HAV IgG (+) (n, %)**  914 (87.4%)  **15-30:** 214 (72.7)  **30-45**: 426 (93.4)  **45-60:** 228 (94.2)  **>60:** 46 (85.1) | **-** | **-** |  | **7/9** |
| **Aşcı et al., (2014)[73], Central Anatolia region, Afyon** | Retrospective study  n:1458 | The aim of this study was to determine the seroprevalence rates of Hepatitis A virus infection in different age groups in the province of Afyonkarahisar | **Age:** *0-57 years* | *May 2*0*12-July 2013* | Electrochemiluminescence method | **Anti-HAV IgG:**  1016 (% 69.68) | ---- | **Anti-HAV IgG:**  **0-2 age:**89(%57.42)  **3-4 age:**46 (%44.23)  **5-6 age:**87(%56.13)  **7-8 age:**129(%66.84)  **9-10 age**:92(%59.35)  **11-12 age:**89 (%64.03)  **13-18 age:**89 (%66.42)  **19-29 age:**95(%86.36)  **30-39 age:**167(%94.35)  **40 and above**:133(% 97.79) | Vaccination history could not be questioned. | **5/9** |
| **Duran and Nazik (2018)[74], eastern anatolia region Bingöl** | Retrospective study  N:19862  n: 4211 children | In this study, we aimed to investigate the seroprevalence of hepatitis A in pediatric age groups in Bingöl province. | **Age:** ≤18 years  **Gender:**  Female: 2337 (%55.5)  Male: 1874 (%44.5) | January 2010 and June 2016 | Chemiluminescent Microparticle Enzyme Immunological Test | **Anti-HAV IgG:** 2444 (%58) | - | **Anti-HAV Ig G:**  **Gender:**  Female:1518( %62.1)  Male: 926 (%37.9)  (p=0,000)  **Age groups:**  **<2 age:** %54.23  **2-6 age:** %38.22  **7-10 age:** %49.58  **11-18 age:** %78.22  **Anti-HAV Ig G:**  **Female**  **<2 age woman:** %63.81  **2-6 age woman:** %36.38  **7-10 age woman:** %53.14  **11-18 age woman:** %82.82  **Male:**  **<2 age man:** %47.20  **2-6 age man:** %39.79  **7-10 age man:** %46.52  **11-18 age man:** %66.88 | ① The vaccine status is not mentioned. | **5/9** |
| **Alıcı et al., (2013)[75], Marmara region İstanbul** | Retrospective study  n: 795 | Hepatitis A seropositivity was investigated in healthy individuals who underwent hepatitis testing for screening purposes. | **Age:** 2-77 years  **Gender:**  Female:504 (%63)  Male:291(%37) | January 2011-December 2012 | ELISA | **Anti HAV IgG:**486(%61) | - | **Anti HAV IgG:**  **Gender:**  Female:  Male**:**  (p=0.169)  **Age groups:**  **0-10 age:** 9 (%21)  **11-20 age:** 24 (%19)  **21-30 age:** 126 (%50)  **31-40 age:** 179 (%81)  **41-50 age:** 95 (%95)  **51-60 age:** 40 (%98)  **>60 age:** 13 (%100)  **Anti HAV IgG:**  **0-10 age**:%21  **11-20 age**: %19  (p=1,000).  **41-50 age**: %95  **>60 age**:%100  (p=1,000).  **0-10 age^a^**  **11-20 age^b^**  **21-30 age^c^**  **31-40 age^d^**  **41-50 age^e^**  **51-60 age^f^**  **>60 age^g^**  e>a,b,c,d; f>a,b,c,d; g>a,b,c,d ; p:0.001 | No Vaccinated | **5/9** |
| **Topal et al., (2011)[76], Marmara region İstanbul** | Cross-sectional  study  n: 319 | The aim of this study is to determine Hepatitis A seroprevalence, rate of hepatitis A vaccination among pre-school age children in urban ûstanbul and emphasize the importance of hepatitis A vaccination | **Age:** 1-6 years  **Gender:**  Female: 121 (%37.9)  Male: 198 (%62.1) | July and October 2008 | ELISA | **Anti-HAV Ig G:** 29 (%9.4) | - | - | Vaccinatio: 11 (%3.4)  ① There was a history of hepatitis A vaccination and these cases were not included in the study when calculating hepatitis A seroprevalence. | **6/9** |
| **Mıngır et al., (2023)[77], Central Anatolia region Afyonkarahisar,** | Crossectional prevelance  N: 8806  n:803 | The aim of our research was to determine the seroprevalence and efficacy of the hepatitis B vaccine routinely administered at birth in students born in 1999 and 2003, and to evaluate the seroprevalence of hepatitis A and hepatitis C. | **Age:** 13 and 17 years  **Gender:**  **Female:** n=454 (%56)  **Male:** 349 (%44) | 2015- 2016 | Chemiluminescent microparticle immunoassay (ABBOTTArchitect İ 2000 SR device) | **Anti-HAV IgG:**n=135(%16.8) | ----- | **Anti-HAV IgG:**  **Gender:**  Female  Male  (p=0.950)  **Age groups:**  **13 age:** 38 (%9.2)  **17 age:** 97 (%24.9)  (p=0.001). | ① Hepatitis A vaccination was not mentioned. | **8/9** |
| **İnci et al. (2020)[78], Black Sea Region, Karabük** | Retrospective  study  n: 4297 | The aim of this study was to investigate the seroprevalence of hepatitis A, hepatitis B and hepatitis C in patients who applied to the Family Medicine out patient clinic at a university hospital. | **Age:**2-91 years  **Gender:**  Female: 2080 (%48.4)  Male: 2217 (%51.6) | January 2016- December 2018 | ELISA | **Anti-HAV IgG:** %57 | -- | --- | ① 2 people vaccinated, rate not given. | **5\8** |
| **Sağlam et al. (2020)[79], Central Anatolia region, Sivas** | Retrospective  study  n: 21.578 | The study aims to determine seroprevalence of hepatitis A virus (HAV) in our region and to determine the prevalence change over years and to evaluate the effects of various factors on prevalence | 0-100 age | 2008-2017 | ELISA | **Anti-HAV IgG:** 10,550 monitored from person's outcome 7940 (%75.3) | --- | **Anti-HAV IgG:**  **Gender:**  Female:75.9  Male:74.6  (p>0.05)  ***Age groups:***  ***0-10 age:*** *668 (%36.5)*  ***11-20 age:****578 (%46.5)*  ***21-30 age:*** *1269*  *(%64.7)*  ***31-40 age:*** *1109 (%94.4)*  ***41-50 age:*** *1135*  *(%99)*  ***51-60 age:*** *1205 (%99.8)*  ***61-70 age:*** *1046*  *(%99.2)*  ***71-80 age:*** *708 (%99.9)*  ***81-90 age:*** *213*  *(%98.6)*  ***91-100 age:*** *9 (%100)*  There is a difference between 0-10 years, 11-20 years, 21-30 years, and other age groups in terms of positivity ratE (p<0.05). | ① The vaccine is not mentioned. | **6/9** |
| **Tüfekçi et al., (2022)[80], Black Sea Region, Kastamonu** | Retrospective study  Anti-HAV IgG studied n:1439 | This study aimed to decide the hepatitis A seroprevalence in all age groups in Kastamonu province, Turkey | **Age:**  Anti-HAV IgG study group age mean: 32.5±19.2 | 2018-2022 | Chemiluminescence microparticle immunoassay method in the Abbott Architect i2000SR | **Anti-HAV IgG:** 1439 patients were examined  837 (%58.2) |  | **Anti-HAV IgG:**  **Gender:**  Female:449(%55.9)  Male: 388(%61.0)  (p:0.052)  **Age groups:**  **0-10 age:**78(%70.1)  **11-20 age**:95(%26)  **21-30 age:**119(%33.7)  **31-40 age:** 130(%71)  **41-50 age:**132(%92.3)  **51-60 age:** 122(%98.4)  **61-70 age:**96(%100)  **≥71 age:**65(%100  The anti HAV IgG positivity increased with the age group (p<0.001). | ① The vaccine status is not mentioned. | **6/9** |
| **Temiz et al. (2015)[81], eastern anatolia region Diyarbakır** | Retrospective  study  n: 3952 | In this study; patients admitted to Diyarbakır Gazi Yaşargil Training and Research Hospital were evaluated retrospectively according to age groups for hepatitis A virus seroprevalence. | Age: 0- 87  Gender:  Female: 1807 (%45.72)  Male: 2145 (%54.28) | January 2010 and December 2014 | Chemiluminescence immune method in Advia Centaur XP (Siemens, Germany) | **Anti HAV IgG:** 1554 patients were examined 1512(%97.3) |  | **Anti HAV IgG:**  **Gender:**  Female:703(%96.80)  Male:809  (%97.70)  *(p:0.367)*  **Age groups:**  **0-10 age ^a^:** 9 (%50)  **11-20 age ^b^:**156( % 91.76)  **21-30 age ^c^:** 416 (%98.34)  **31-40 age ^d^:** 323(% 99.07)  **41-50 age ^e^:** 222(%99.10)  **51-60 age ^f^:** 145(%98.63)  **≥61age ^g^:** 241(%97.96)  a<b,c,d,e,f,g  (p=0.015). | ① Vaccination history could not be questioned in this study. | **6/9** |
| **Genç et al. (2006)[82], Central Anatolia Region, Ankara** | Retrospective study  n: 736 | The purpose of this study was to determine the seroprevalence of Hepatitis A infection among 2-6 year-old children of low socioeconomic class families. | **Age:** 2-6 age  **Gender:**  Female:352  Male: 384 | September 2002 and May 2003 | Microparticle enzyme immunoassay (AXSYM instrument) and hepatitis A virus antibody samples (Abbott Laboratory)ı | **Anti-HAV IgG:**26(%3.5) |  | **Anti-HAV IgG:**  **Gender:**  Female (42-72 months):9  Male (42-72 months):17  **Age groups:**  **24-30 months:** 0 (%0)  **30-36 months:** 0 (%0)  **36-42 months:** 0 (%0)  **42-48 months:** 2(%0.27)  **48-54 months:**3(%0.4)  **54-66 months:**6(%0.8)  **66-72 months:**15(%2) | ① There was no history of hepatitis A vaccination in any of the children. | **7\9** |
| **Akman et al. (2020)[83], Central Anatolia Region Ankara** | Retrospective study  n: 3238 | The study aimed to describe changes in the seroprevalence of hepatitis A virus from the prevaccine era (2012) to the post-vaccine era (2018) in different age groups | 0-18 age children: 2820  ≥18 adult::418 | 2013-2018 | Microplate-based enzyme-linked immunosorbent assay (Etimax-3000, Diasorin) | ***-----*** | ----- | ***Anti-HAV IgG:***  Children group:1147/2820  (%40.7)  **Age groups**:  ≤2 years:%60.5  2–6 years:%42.1  7–11 years:%68.8  12–18 years:%67.3  19–24 years:%55.4  25 years and older:%55.9  children group (male): 1565/2820 (%55.5)  Between 2013 and 2018, the increase in the number of seropositive individuals in group 2 (p<0.01), and the decrease in groups 3 and 4 were statistically significant (p=0.028, p<0.01).  ***Anti-HAV IgG:***  ≤2 age (2013): 52 (%60.5)  ≤2 age (2014):83(%63.8)  ≤2 age (2016):30(%51.7)  ≤2 age (2017):51(%55.4)    ≤2 age (2018):25(%71.4)  **p:0.27**  ***Anti-HAV IgG:***  2-6 age (2013)^a^: 54 (%35.3): cde  2-6 age (2014)^b^:88(%49.4):de  2-6 age (2016)^c^:34(%72.3): ae  2-6 age (2017)^d^:83(%74.1):abe    2-6 age (2018)^e^:41(%95.3):abcd  **p<0.001**  ***Anti-HAV IgG:***  7-11 age (2013):60 (%38)  7-11 age (2014):62(%27)  7-11 age (2016):34(%31.2)  7-11 age (2017):40 (%25.5)  7-11 age (2018):38 (%39.6)  **p:0.028**  ***Anti-HAV IgG:***  12-18 age (2013)^a^:86(%44.3):cde  12-18 age (2014)^b^:136 (%37.8):cd  12-18 age (2016)^c^:32(%22.2):ab  12-18 age (2017)^d^:77(%27.2):ab  12-18 age (2018)^e^:41(%26.5):a  **p:**<**0.001**  ***Anti-HAV IgG:***  19-24 age (2013):5(%50)  19-24 age (2014):11(%50)  19-24 age (2016):7(%38.9)  19-24 age (2017):8(%33.3)  19-24 age (2018):10(%55.6)  **p: 0.60**  ***Anti-HAV IgG:***  >25 age (2013):49(%80.3)  >25 age (2014):54(%71.1)  >25 age (2016):36(%75)  >25 age (2017):62(%72.1)  >25 age (2018):40(%72.7)  **p:0.76** | ① Vaccination status was not mentioned. | **7/9** |
| **Kolancal et al. (2017)[84], Marmara region İstanbul** | Cross-sectional study  n:400 | In this study; the aim was to evaluate the seroprevalence of hepatitis A virus (HAV) in 2-16 year age group, and the rate of hepatitis A vaccination | Age:2-16 years  Female: 177 (%44.3)  Male:223(%55.8)  2-5 age: 78 (%19.5)  6-16 age: 322 (%80.5) | July 2016 and September 2016 | Enzyme-linked immunosorbent assay and the measurements were made in mIU/ mL (Architect Systems and Abbott Diagnostics Division, the USA) | **Anti-HAV IgG:**  109 (%27.3)  5.9% of children who were unvaccinated. | ----- | **Anti-HAV IgG:**  2-5 age: 46 (59%)  6-16 age: 63(19.6%) | Vaccination:44 (%11)  Vaccination: 2-5 age: 56.4% (n=44)  Unvaccinated: 356 (%89)  Unvaccinated:  2-5 age: 43.6%  Unvaccinated:  6-16 age:322 | **6\9** |
